# Peptidoglycan Branched Stem Peptides Contribute to *Streptococcus pneumoniae* Virulence by Inhibiting Pneumolysin Release

**DOI:** 10.1371/journal.ppat.1004996

**Published:** 2015-06-26

**Authors:** Neil G. Greene, Ana R. Narciso, Sergio R. Filipe, Andrew Camilli

**Affiliations:** 1 Graduate Program in Molecular Microbiology, Sackler School of Graduate Biomedical Sciences, Howard Hughes Medical Institute, and Department of Molecular Biology and Microbiology, Tufts University School of Medicine, Boston, Massachusetts, United States of America; 2 Laboratory of Bacterial Cell Surfaces and Pathogenesis, Instituto de Tecnologia Quimica e Biologica, Universidade Nova de Lisboa (ITQB-UNL), Oeiras, Portugal; The University of Alabama at Birmingham, UNITED STATES

## Abstract

*Streptococcus pneumoniae* (the pneumococcus) colonizes the human nasopharynx and is a significant pathogen worldwide. Pneumolysin (Ply) is a multi-functional, extracellular virulence factor produced by this organism that is critical for pathogenesis. Despite the absence of any apparent secretion or cell surface attachment motifs, Ply localizes to the cell envelope of actively growing cells. We sought to characterize the consequences of this surface localization. Through functional assays with whole cells and subcellular fractions, we determined that Ply activity and its release into the extracellular environment are inhibited by peptidoglycan (PG) structure. The ability of PG to inhibit Ply release was dependent on the stem peptide composition of this macromolecule, which was manipulated by mutation of the *murMN* operon that encodes proteins responsible for branched stem peptide synthesis. Additionally, removal of choline-binding proteins from the cell surface significantly reduced Ply release to levels observed in a mutant with a high proportion of branched stem peptides suggesting a link between this structural feature and surface-associated choline-binding proteins involved in PG metabolism. Of clinical relevance, we also demonstrate that a hyperactive, mosaic *murMN* allele associated with penicillin resistance causes decreased Ply release with concomitant increases in the amount of branched stem peptides. Finally, using a *murMN* deletion mutant, we observed that increased Ply release is detrimental to virulence during a murine model of pneumonia. Taken together, our results reveal a novel role for branched stem peptides in pneumococcal pathogenesis and demonstrate the importance of controlled Ply release during infection. These results highlight the importance of PG composition in pathogenesis and may have broad implications for the diverse PG structures observed in other bacterial pathogens.

## Author Summary

Pneumolysin (Ply) is a protein toxin produced by *Streptococcus pneumoniae* that contributes to the ability of this organism to cause invasive disease. Release of this protein from the bacterial cell is necessary for many of its functions but the underlying mechanisms driving this process are not well characterized. Previous research demonstrated that Ply localizes to the cell wall compartment. Here, we address the consequences of this localization and reveal a role for the major cell wall structural component, peptidoglycan, in inhibiting Ply activity and release into the extracellular environment. Peptidoglycan is an essential, mesh-like sac that encases the cell, and alterations affecting its composition lead to differences in the amount of Ply released. How molecules interact with and traverse through the restrictive matrix of the cell wall and its associated structures is incompletely understood, particularly with respect to protein secretion and surface attachment. Our results argue that proper maintenance of cell wall-associated Ply is dependent on surface architecture and may be critical for *S*. *pneumoniae* pathogenesis.

## Introduction

*Streptococcus pneumoniae* (the pneumococcus) is a Gram-positive commensal of the human nasopharynx. Though asymptomatic, nasal carriage is considered a prerequisite for the establishment of invasive pneumococcal disease [1]. The pneumococcus is an extracellular pathogen that elaborates a multitude of virulence determinants that contribute to the pathogenesis of invasive pneumococcal diseases such as otitis media, pneumonia, meningitis and bacteremia, depending on the site of infection. Pneumolysin (Ply) is one such conserved, multi-functional virulence factor [2]. As a member of the cholesterol-dependent cytolysin family of pore-forming toxins, Ply is cytotoxic to a variety of eukaryotic host cells [3–5]. Additional activities attributed to Ply include complement activation, induction of host cell signaling cascades, and stimulation of a diverse array of cytokines [6–9].

Ply must be extracellular to carry out the aforementioned functions, however, unlike all other Gram-positive cholesterol-dependent cytolysins, Ply lacks a canonical N-terminal signal peptide commonly associated with Sec-mediated secretion. Additionally, Ply does not encode any of the currently known motifs necessary for cell envelope attachment [10]. Despite these observations, previous studies have demonstrated that Ply is present in culture supernatants [11] and the cell wall compartment during growth [12]. Furthermore, these studies ruled out a role for the major pneumococcal autolysin LytA in this process, suggesting that autolysis alone could not account for extracellular Ply. Therefore, Ply export from the cytoplasm to the cell envelope has been proposed to occur via an active process that remains to be discovered [13].

A defining characteristic of the Gram-positive cell envelope is a thick layer of peptidoglycan (PG) that encompasses the cell. PG is a rigid, yet dynamic, macromolecule that confers the characteristic shape of bacterial cells and provides protection against lysis from turgor pressure. The basic structure of PG is conserved and consists of a mesh-like network of glycan strands situated circumferentially around the cell composed of alternating N-acetylglucosamine and N-acetylmuramic acid residues crosslinked through short peptides emanating from the latter sugar moiety [14]. Variation among PG types largely exists at the level of stem peptide composition and the penicillin-binding protein (PBP)-catalyzed transpeptidation reactions that serve to generate crosslinks between them [15]. Pneumococcal PG is characterized by a combination of linear and branched stem peptides [16]. Formation of branched stem peptides occurs through addition of a dipeptide branch on the third position lysine residue of a nascent PG precursor molecule during the membrane-associated steps of PG biosynthesis. This activity is catalyzed by two gene products encoded within the *murMN* operon [17–19]. Crosslink formation between the dipeptide branch and an adjacent stem peptide results in formation of a crossbridge, thus incorporating branched stem peptides into the existing PG network. Therefore, *murMN* not only affects the peptide composition of PG but also is directly involved in the structural integrity of the mature molecule.

Although the exact role of branched stem peptides in pneumococcal biology is ill-defined, it has been shown that *murMN* expression is necessary for penicillin resistance (Pen^R^) [20]. Furthermore, PG isolated from Pen^R^ strains displays a marked shift towards a high proportion of branched stem peptides [21]. This shift is attributed to significant divergence within the coding region of *murM* [22,23] with some mutations conferring increased catalytic activity to MurM [18]. Thus, *murMN* is necessary for Pen^R^ and mosaic *murM* alleles are commonly found in Pen^R^ isolates; however, expression of *murMN* is not sufficient for Pen^R^ [24]. The exact association between Pen^R^ and *murMN* remains unclear, but it has been hypothesized that expression of low-affinity PBPs, which confer Pen^R^, demonstrate altered substrate specificity [21], which may drive the selective pressure for mosaic *murM* alleles capable of generating higher quantities of the preferred branched stem peptide substrate.

In addition to its protective role, PG serves as a scaffold to which numerous secreted molecules are anchored including, but not limited to, a diverse array of proteins that serve a variety of functions for pneumococcal physiology and pathogenesis. Attachment of these proteins can be direct, as in the case of sortase-mediated covalent linkage to PG, or indirect, such as the non-covalent interaction between choline-binding proteins and the PG-linked wall teichoic acids [25]. Despite an extensive knowledge of protein export and the mechanisms responsible for their physical tethering to the cell surface, little is known about the traversal of secreted proteins not destined for attachment to the cell surface during PG maturation. Given its mesh-like structure, it has been postulated that PG acts as a barrier to the release of secreted proteins [26]. In support of this model, early observations in *Bacillus amyloliquefaciens* demonstrated that washed cells continue to release the secreted protein α-amylase even after inhibition of protein synthesis and Sec-mediated secretion, suggesting the existence of a surface-associated reservoir of this protein [27].

The functional consequences of Ply localization to the cell envelope remain unexplored. In this study, we tested the hypothesis that surface-associated Ply is active and contributes to pneumococcal pathogenesis. Our results indicate that Ply activity and release into the extracellular milieu is inhibited by PG structure. Ply release from the cell appears to be dependent on both the incorporation of branched stem peptides in the PG layer and the action of surface-bound choline-binding proteins. Finally, we demonstrate the importance of appropriate Ply release during infection and the role of branched stem peptides in this process.

## Results

### Native cell wall structure inhibits Ply activity and release from the cell

**Fig 1:**
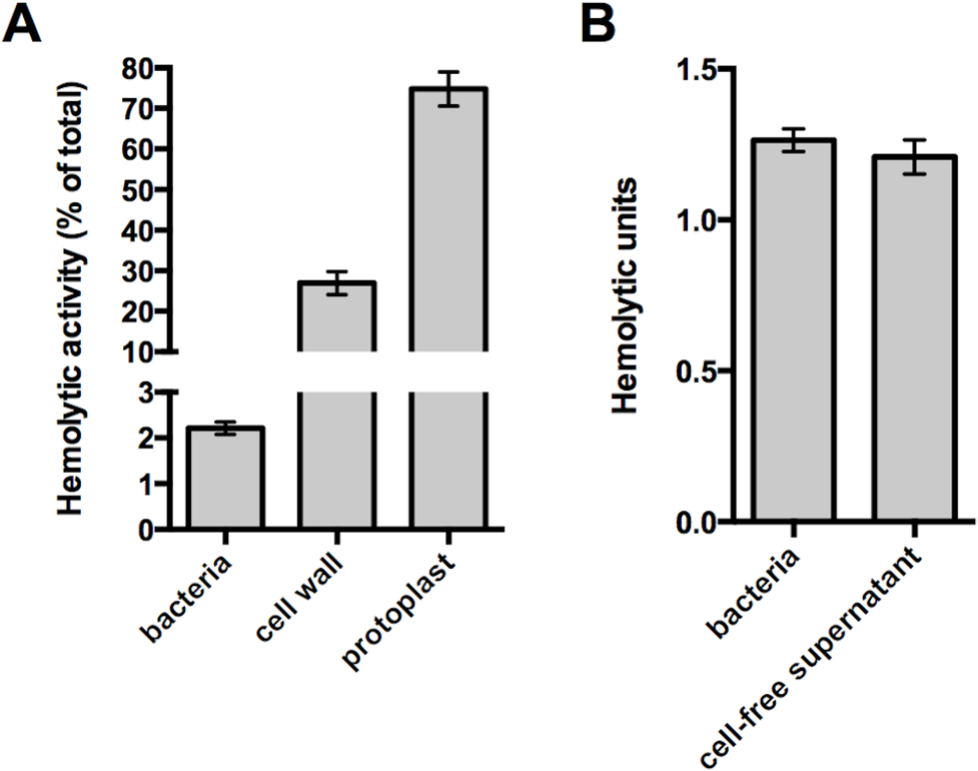
The native cell wall inhibits Ply activity and release. **(A)** Washed whole cells, subcellular fractions (cell wall and protoplast), or sonicated cell lysates of wildtype pneumococcus were tested for hemolytic activity as described in *Materials and Methods*. Protoplasts were lysed by sonication prior to the experiment. The hemolytic activity of each sample is represented as a percent of the total as determined for the sonicated cell lysate. **(B)** To determine if the activity of intact cells is due to cell-associated or secreted Ply, washed whole cells were incubated with SRBCs or buffer alone for one hour. Cells incubated with buffer alone were pelleted and the cell-free supernatant was removed and tested for hemolytic activity as described in *Materials and Methods*. Of note, Ply is the only active hemolysin under these conditions; deletion of *ply* completely abolishes hemolytic activity. Columns represent the mean and error bars denote SEM of at least four **(A)** or two **(B)** biological replicates.

To assess the amount of functional, surface-accessible Ply compared to the amount present in the cell wall compartment and cytoplasm, hemolysis assays were performed with washed pneumococci. While washed bacteria accounted for only two percent of the total Ply-dependent hemolytic activity, the isolated cell wall fraction harbored ~30% of the total activity (Fig 1A). This discrepancy suggests that the native cell wall structure is capable of masking Ply exposure on the cell surface, and its liberation is dependent on enzymatic digestion of the PG layer. Protoplasts exhibited the highest hemolytic activity (Fig 1A), indicating that the majority of Ply is retained in the cytoplasm and/or membrane fraction. The activities observed for both cell wall and protoplast fractions correlate well with the amount of Ply detected in each fraction by Western blot analysis [12], which we confirm here (S1 Fig, wt).

Given that the washed cell sample demonstrated hemolytic activity, we sought to determine if the Ply responsible for this hemolysis remains surface-associated or is released from the cell surface. To address this question, paired whole cell samples were incubated with sheep red blood cells or buffer alone for a fixed amount of time. After this incubation, the bacterial cells were removed from the buffer alone sample by centrifugation and the cell-free supernatant was tested for hemolytic activity. The cell-free supernatant harbored the same activity as the whole cell sample indicating that all of the hemolysis observed with washed cells is due to Ply that has dissociated from the cell surface (Fig 1B). Given this, we wondered whether the low amount of Ply release relative to the total Ply present in the cell wall compartment could be explained by binding of Ply to the PG layer. Consistent with the apparent absence of any PG-binding motif, we were unable to detect binding of Ply to purified PG over a range of protein and PG concentrations using a pull-down assay (S2 Fig). Collectively, these data suggest that cell envelope-associated Ply is either not surface-exposed or is somehow inhibited from functioning while still cell-associated.

### Branched stem peptides in the PG structure inhibit Ply release

**Fig 2:**
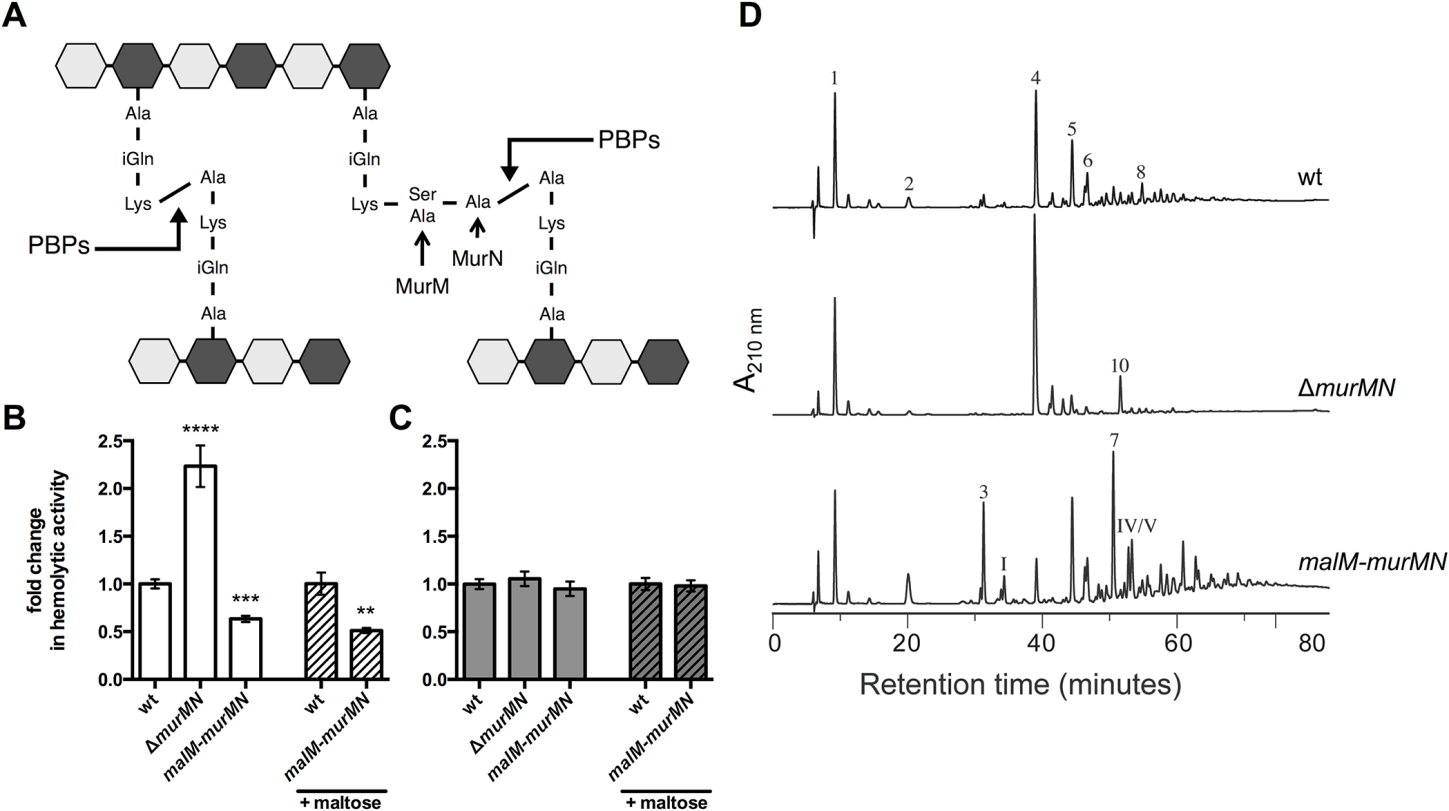
Ply release is altered by *murMN*-dependent changes in PG composition. **(A)** Diagram of the basic pneumococcal PG structure highlighting the role of MurM and MurN. PG is a heteropolymer of glycan strands that alternate between N-acetylglucosamine (NAG, light grey hexagon) and N-acetylmuramic acid (NAM, dark grey hexagon) residues crosslinked through short peptides that stem from the NAM moiety. Stem peptides can be linear or branched; the latter form is dependent on MurM and MurN. Penicillin-binding proteins (PBPs) catalyze the crosslinking reaction that links adjacent stem peptides. **(B-C)** Hemolytic activity of whole cells **(B)** or sonicated lysates **(C)** of wildtype (wt) and mutants either lacking *murMN* or carrying a second copy of *murMN* under the control of the maltose-inducible promoter were grown with or without 0.8% maltose as indicated. Data are presented as the mean fold change in hemolytic activity compared to the wt in each condition ± SEM of at least four biological replicates. ** p < 0.01, *** p < 0.0005, **** p < 0.0001, Student’s t-test. **(D)** RP-HPLC analysis of stem peptides isolated from purified PG of the wt, *murMN* deletion, and *malM-murMN* overexpression strains. PG purification, stem peptide removal, and detection by RP-HPLC were performed as described in *Materials and Methods*. Peptides were detected by their absorption at 210 nm. The peptide structures of indicated peaks are outlined in S4 Fig.

Stem peptide composition within pneumococcal PG displays a high degree of heterogeneity through the cell cycle and this diversity is further extended between different pneumococcal strains [23,28]. One feature contributing to this variation is the presence of both linear and branched stem peptides, the latter of which are formed by products of the *murMN* operon (Fig 2A) [17]. MurM and MurN act sequentially to catalyze the tRNA-dependent addition of a dipeptide onto the lysine residue of a PG precursor molecule; MurM acts first to add either a serine or alanine residue, which provides the substrate for the MurN-dependent addition of an alanine [17–19]. Deletion of *murMN* has no effect on growth *in vitro* or the apparent amount of crosslinking within PG, yet manipulation of the *murMN* operon causes drastic changes in the composition of stem peptides and the type of crosslinks that connect them [17,24]. Therefore, we reasoned that studies of *murMN* would allow us to determine the effects of PG composition and structure on Ply release.

Deletion of the *murMN* operon caused a two-fold increase in Ply release as measured by hemolytic activity of whole cells when compared to the wildtype (wt) (Fig 2B, Δ*murMN*), suggesting a role for the products of this operon in controlling Ply release. Overexpression of *murM* has previously been shown to favor production of branched stem peptides at the expense of linear stem peptides [29]. To test if increasing the proportion of branched stem peptides would yield the opposite phenotype of Δ*murMN*, we created a merodiploid strain carrying a second copy of *murMN* downstream of a maltose-inducible promoter [30]. In the absence of inducer, *murMN* overexpression caused a decrease in hemolytic activity of washed cells to the same magnitude as the *murMN* deletion (Fig 2B, *malM-murMN*) suggesting that basal expression from the maltose promoter is sufficient to increase *murMN* transcript levels, which was supported by quantitative reverse transcription-PCR (qRT-PCR) (S3 Fig). Supplementation of the growth medium with inducer did not further augment this decrease (Fig 2B) despite increased expression of the entire operon compared to growth without inducer as measured by qRT-PCR (S3 Fig). Thus, changes in *murMN* expression are associated with differential Ply release from the cell.

To rule out the possibility that genetic manipulation of the *murMN* operon caused alterations in Ply production or stability, which could account for the phenotypes observed we also measured the hemolytic activity of cell lysates. Mutants lacking or overexpressing the *murMN* operon all harbored the same total hemolytic activity as wt (Fig 2C), supporting the notion that all strains tested contain the same amount of Ply during the course of the experiment. Furthermore, we determined that Ply localization to the cell wall compartment was unaffected by deletion or overexpression of *murMN* (S1 Fig), indicating that the phenotype observed for washed, whole cells is not due to defects in Ply production or trafficking to the cell surface. Experiments described later will demonstrate that modest changes in the specific localization of Ply can have profound consequences.

In order to verify that Δ*murMN* and *malM-murMN* exhibited distinct stem peptide profiles, we purified PG from each strain and analyzed its peptide composition by reversed phase-high performance liquid chromatography. The wt strain was included as a control. As depicted in Fig 2D, wt PG contained both linear and branched stem peptides. Peptide structures of assigned peaks can be found in S4 Fig. Within the monomeric species, the linear tripeptide (peak 1) represented 20.8% of the total peptide material analyzed compared to 3.2% for the branched counterparts (peaks 3 and I) (Table 1). However, dimers containing at least one branched structure (peaks 5, 6, 7, IV, V, VI) were modestly increased compared to the directly crosslinked linear dimer (peak 4) (Table 1). These data demonstrate that branched stem peptides can be found throughout wt PG in a manner similar to that observed in PG from other penicillin-sensitive (Pen^S^) laboratory strains [20].

**Table 1:** Stem peptide composition of select *S*. *pneumoniae* strains.^a^

Peak	Peptide characteristics		wt^b^	Δ*murMN*	*malM-murMN*	*murMN* ^TIGR4^ ^c^	*murMN* ^R36A^ ^c^	*murMN* ^Pen6^ ^c^
**1**	Linear	Monomer	20.8 (0.31)	23.7 (0.97)	11.8 (0.20)	20 (1.01)	20.6 (0.38)	6.9 (0.41)
**2**	Linear	Monomer	4.1 (0.09)	1.6 (0.11)	7.1 (0.36)	4.8 (0.95)	2.9 (0.16)	9.9 (0.65)
**3**	Branched	Monomer	2.3 (0.14)	0	10.6 (0.17)	2.8 (0.76)	2.9 (0)	7 (1.51)
**I**	Branched	Monomer	0.9 (0.16)	0	3.0 (0.29)	1.1 (0.53)	1.4 (0)	6.2 (1.22)
**4**	Linear	Dimer	24.7 (0.21)	57.2 (1.48)	5.2 (0.03)	21.6 (1.15)	30.7 (0.30)	2 (0.28)
**5**	Branched	Dimer	15 (0.04)	4.8 (0.16)	12.8 (0.43)	15.3 (1.44)	12.9 (0.26)	4.4 (0.17)
**6a**	Branched	Dimer	3.9 (0.10)	1.1 (1.59)	4 (0.22)	3.7 (0.45)	3.5 (0.12)	1.1 (0.05)
**6b**	Branched	Dimer	7.7 (0.10)	1.3 (1.9)	5.8 (0.10)	8.1 (0.37)	6.7 (0.10)	5.5 (0.22)
**7**	Branched	Dimer	4.3 (0.02)	0	17 (0.75)	5.5 (0.47)	2.9 (0.04)	12.5 (0.29)
**10**	Linear	Trimer	2.9 (0)	8.3 (0.40)	0.8 (0.51)	2.6 (0.16)	3.7 (0.01)	1.6 (0.15)
**IV**	Branched	Dimer	1.8 (0.04)	0	5.6 (0.15)	2.3 (0.09)	1.5 (0.03)	10.9 (0.03)
**V**	Branched	Dimer	2.5 (0.04)	0.6 (0.88)	7.5 (0.10)	3.3 (0.12)	2.2 (0.03)	14.5 (0.29)
**8**	Branched	Trimer	4.6 (0.01)	0	2.7 (0.09)	4 (0.69)	4.5 (0.06)	1.4 (0.09)
**VI**	Branched	Dimer	1.3 (0.09)	0.7 (0.95)	2.6 (0.06)	1.7 (0.03)	1.4 (0.08)	14.7 (1.83)
**9**	Branched	Trimer	3.1 (0.08)	0.5 (0.76)	3.4 (0.37)	3.3 (0.23)	2.3 (0.07)	1.2 (0.06)
**Total**			100	100	100	100	100	100
**Branched peptides (%)**			47.6 (0.01)	9.1 (2.74)	75.1 (0.69)	51.1 (0.65)	42.0 (0.07)	79.6 (0.10)
**Monomers**			28.1 (0.08)	25.4 (0.85)	32.6 (0.61)	28.7 (0.68)	27.8 (0.22)	30.0 (2.49)
**Branched monomers (%)**			11.6 (1.03)	0	42.0 (0.63)	13.7 (4.80)	15.2 (0.14)	43.9 (5.45)
**Oligomers**			71.9 (0.08)	74.6 (0.85)	67.4 (0.61)	71.3 (0.68)	72.2 (0.22)	70.0 (2.49)
**Branched oligomers (%)**			61.7 (0.33)	12.2 (3.53)	91.1 (0.89)	66.2 (1.52)	52.3 (0.26)	94.8 (0.38)
**Crossbridged oligomers** ^**d**^ **(%)**			50.9 (0.48)	10.4 (1.01)	82.5 (1.11)	54.8 (1.93)	43.1 (0.37)	86.9 (0.34)

^a^ Each peak corresponds to a specific peptide structure as depicted in S4 Fig. Values represent the mean abundance of the indicated species relative to the total amount of peptide material analyzed and numbers within parentheses denote the standard deviation of two independent experiments.

^b^ TIGR4 strain

^c^ The *murMN* coding regions and intervening sequence from each strain (TIGR4, R36A, Pen6) were introduced into Δ*murMN*, replacing the chloramphenicol resistance marker at the native chromosomal locus

^d^ A crossbridge is defined as a crosslink that directly incorporates the branch structure (covalent linkage between alanine from branch and fourth position alanine of adjacent peptide)

In contrast to wt, PG from Δ*murMN* was characterized by a virtual loss of branched peptides and a concomitant overrepresentation of linear peptides (Fig 2D and Table 1). In particular, linear peptides accounted for 90.9% of the total material analyzed from this strain, with the directly crosslinked dimer being the most abundant at 57.2% (Table 1). Strikingly, overexpression of *murMN* caused a drastic shift in the PG stem peptide profile compared to both wt and Δ*murMN* (Fig 2D). The abundance of monomers containing a branched structure (peaks 3 and I) increased to 13.6%, approximately four-fold higher than in the wt (Table 1). Furthermore, the enrichment in branched peptides was particularly noticeable in the crosslinked material of this strain. Dimers containing a branched structure (peaks 5, 6, 7, IV, V, VI) represented 55.3% of the total peptide material at the expense of the linear dimer (peak 4), which decreased five-fold compared to the wt (Table 1). Additionally, there was a near complete loss in the linear trimer (peak 10) in *malM-murMN* (Table 1). Given that the stem peptide profile from *malM-murMN* was prepared from this strain grown without inducer, these data indicate that a modest ~1.5-fold overexpression of *murMN* is sufficient to cause profound changes to the PG layer (S3 Fig). The peptide profiles depicted in Fig 2D are wholly consistent with previously published results from *murMN* deletion and overexpression mutants in diverse strain backgrounds [20,29]. Taken together, these data strongly support a role for branched stem peptides in limiting Ply release into the extracellular environment.

### Choline-binding proteins contribute to Ply release and are sensitive to branched stem peptides

PG is a dynamic molecule that is continuously remodeled during growth and division through the activity of numerous enzymes collectively referred to as PG hydrolases. These factors catalyze PG degradation by cleaving distinct bonds within this structure and, consequently, if not properly regulated can result in cell lysis [31]. Given the association between PG and Ply observed thus far, we hypothesized that Ply release could be due to cleavage of the cell wall by PG hydrolases. To address this possibility, we took advantage of the fact that the major pneumococcal PG hydrolases contain choline-binding domains and are therefore displayed on the cell surface by virtue of binding to the choline residues of teichoic acids [2]. This interaction is non-covalent and can be disrupted by the addition of exogenous choline, causing release of choline-binding proteins (CBPs) from the cell surface [32]. Thus, choline treatment would simultaneously remove multiple PG hydrolases (e.g. LytA, LytB, LytC, CbpD) from the cell surface as well as other CBPs that harbor distinct functions, allowing us to assess the contribution of this entire subset of proteins to Ply release.

**Fig 3:**
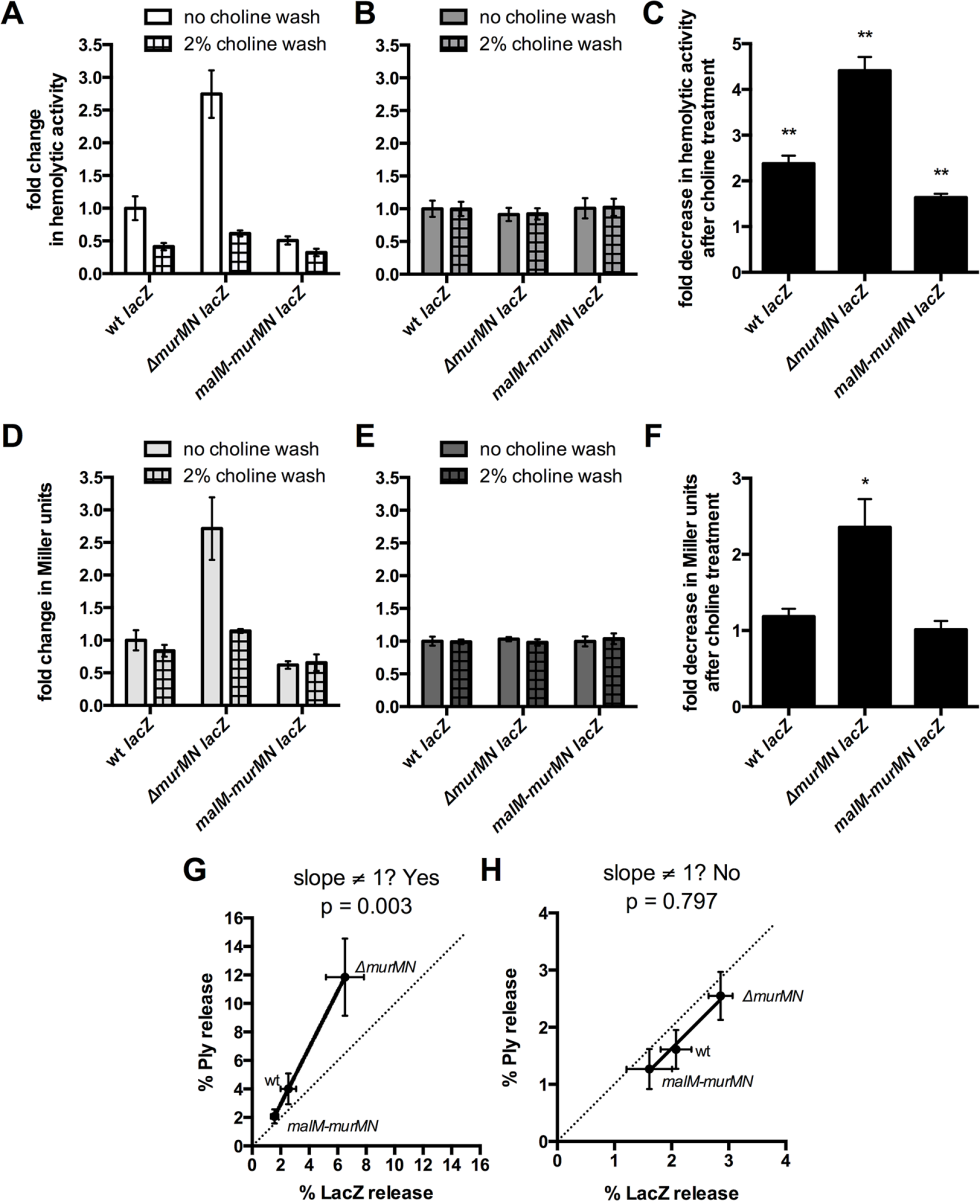
Choline-binding proteins contribute to Ply and LacZ release in a *murMN*-dependent manner. The endogenous β-galactosidase gene, *bgaA*, was replaced with *E*. *coli lacZ* expressed from a constitutive promoter in wt, Δ*murMN*, and *malM-murMN* and the resulting strains were tested for hemolytic **(A-B)** and β-galactosidase **(D-E)** activity after incubation without or with 2% choline chloride. Strains were tested for both activities as whole cells **(A, D)** and sonicated lysates **(B, E)**. The fold decrease in hemolytic **(C)** and β-galactosidase **(F)** activity between the choline-treated and control groups of each strain was quantified. Columns represent the mean and error bars denote SEM of four biological replicates. In **(A-B, D-E)** the data are presented as the fold change in measured activity compared to the wt no choline wash group. * p < 0.05, ** p < 0.01, One sample t test compared to a value of 1.0, which indicates no change. **(G-H)** Percent Ply and LacZ release was calculated for each strain in both conditions by dividing the whole cell activity in **(A)** or **(D)** by the sonicated lysate activity in **(B)** or **(E)**, respectively. The calculated values were plotted against each other for the control **(G)** and choline-treated **(H)** groups such that each symbol represents a different strain with error bars indicating SEM. Data were fit to a straight-line model and the resulting slope was compared to a hypothetical value of 1.0, which represents equal change between the two variables measured and is indicated by the dotted line.

Prior incubation with 2% choline decreased the hemolytic activity of supernatants prepared from whole cells of wt, Δ*murMN*, and *malM-murMN* compared to the no choline wash control (Fig 3A). By contrast, choline treatment had no effect on the total hemolytic activity from cell lysates of either strain tested (Fig 3B). This suggests that the ability to release Ply is dependent on the presence of CBPs on the cell surface. Strikingly, the magnitude by which Ply release decreased was dependent on the strain background tested (Fig 3C). The most pronounced change was observed in Δ*murMN*, which decreased approximately four-fold after the choline wash relative to the control sample (Fig 3C). By comparison, wt experienced a two-fold drop in Ply release while *malM-murMN* was modestly, yet significantly, reduced by 1.5-fold (Fig 3C). These results suggest that CBPs contribute to Ply release but this effect is sensitive to the proportion of branched stem peptides within PG.

Given that Ply is present in both the cell wall compartment and the cytoplasm, we wanted to address the origin of the released Ply observed in washed, whole cells. It is formally possible that the hemolytic activity of whole cells could be explained by specific Ply release from the cell wall fraction due to PG cleavage. Alternatively, the activity could be explained by lysis of a sub-population of cells, which would release Ply from both the cell wall and the cytoplasm. To distinguish between these possible explanations, we reasoned that we could test for the presence of a strictly cytoplasmic marker in addition to Ply. If Ply release is the result of lysis, then we should also detect the cytoplasmic marker; if lysis is not the primary mechanism, there should be enrichment in Ply over the cytoplasmic marker. A commonly used, robust and easily detectable cytoplasmic marker is β-galactosidase, encoded by *E*. *coli lacZ* [33]. Therefore, we replaced the coding region of the endogenous β-galactosidase, *bgaA*, with that of *lacZ* under the control of a constitutive promoter in the wt, Δ*murMN*, and *malM-murMN* strains and tested for the presence of LacZ in whole cells and sonicated lysates with and without choline treatment.

Miller assays to detect β-galactosidase activity were performed on the same samples used to measure hemolytic activity depicted in Fig 3A and 3B. Supernatants from whole cells of Δ*murMN* expressing *lacZ* contained approximately twice as much β-galactosidase activity as wt or *malM-murMN* (Fig 3D, no choline wash). Overexpression of *murMN* resulted in a modest decrease in LacZ release from whole cells that was not significantly different from wt (Fig 3D, no choline wash). Interestingly, choline treatment abolished the two-fold increase in β-galactosidase activity observed in supernatants from whole cells of Δ*murMN*, reducing it to levels comparable to that of the untreated wt sample (Fig 3D). Importantly, neither expression of *murMN* nor choline treatment affected the total β-galactosidase activity of cell lysates (Fig 3E), indicating a similar amount of LacZ was present in each strain and condition tested. Therefore, LacZ release increased upon deletion of *murMN* in a manner dependent on CBPs, whereas wt and *malM-murMN* had similar levels of LacZ release that are unaffected by CBPs (Fig 3F).

To determine whether there was any relationship between the amount of Ply and LacZ released from washed cells of each strain, we calculated the percentage of each protein released from whole cells and fit the data to a straight-line model. By this metric, a slope of 1 is indicative of an equal proportion of Ply and LacZ in the supernatants, which would suggest lytic release of each protein from the cytoplasm. As shown in Fig 3G, there was enrichment in the amount of Ply present in each sample, particularly for Δ*murMN lacZ*, as determined by skew towards the y-axis, and the resulting slope was significantly different than 1. However, a similar analysis performed with the choline-treated samples revealed a slope that was not significantly different than 1 (Fig 3H). Taken together, these data suggest that CBPs contribute to Ply release primarily from the cell wall compartment, but this effect is dependent on the proportion of branched stem peptides in the PG layer. Additionally, some but not all of the Ply release observed in each strain can be attributed to cell lysis. This is particularly apparent for Δ*murMN*, which releases half as much LacZ as Ply in a CBP-dependent manner (Fig 3G). However, in the absence of CBPs, Ply release can be solely attributed to lysis, presumably due to the actions of other PG hydrolases within the cell (see Discussion).

### Repair of Δ*murMN* with distinct *murMN* alleles alters Ply release and PG composition

**Fig 4:**
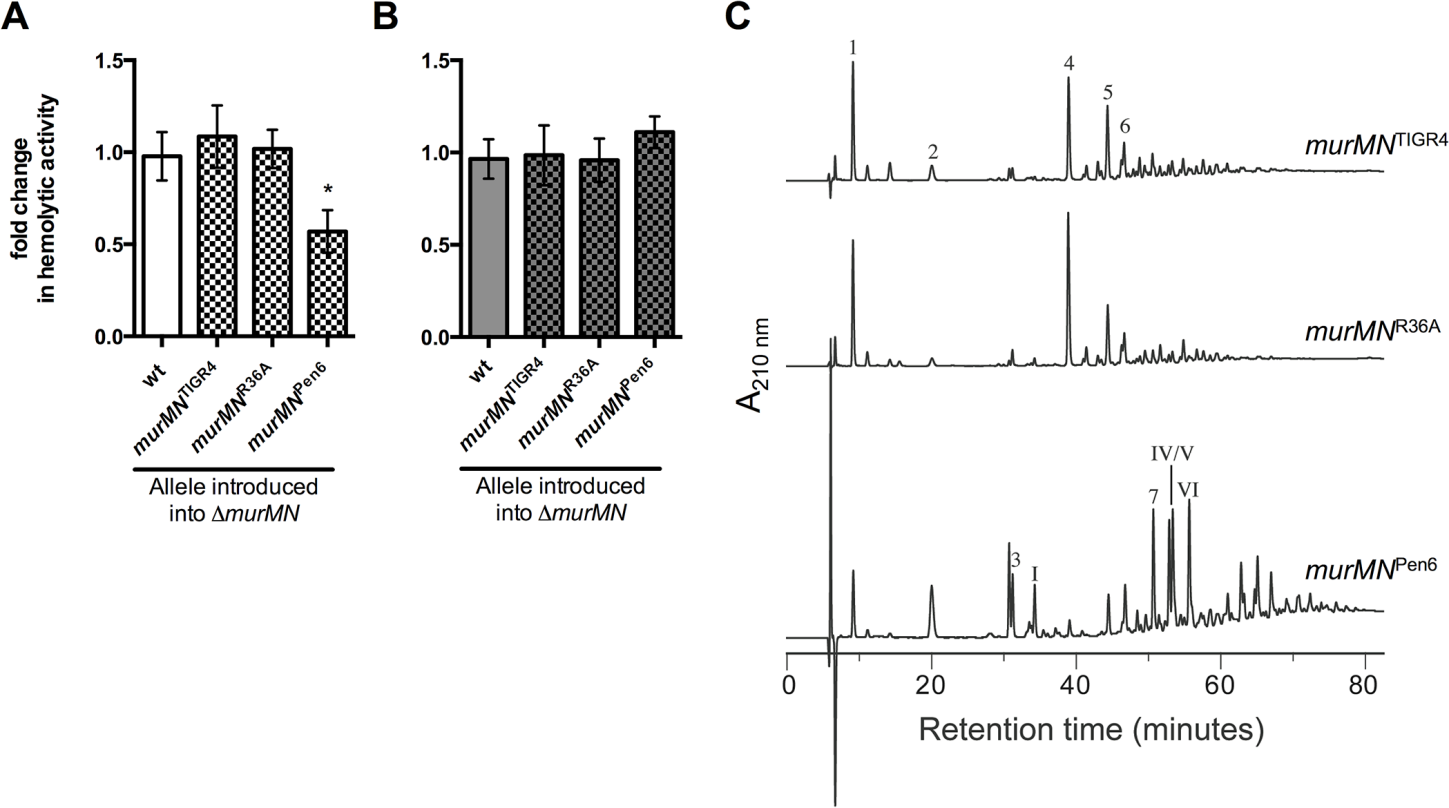
Increased Ply release observed upon *murMN* deletion can be differentially restored by variant *murMN* alleles. Hemolytic activity of whole cells **(A)** or sonicated lysates **(B)** of marked Δ*murMN* strains expressing the *murMN* operon from wt (*murMN* ^TIGR4^), R36A (*murMN* ^R36A^) or Pen6 (*murMN* ^Pen6^). The wt chromosomal *murMN* promoter controls expression of each allele. Data are presented as the mean fold change in hemolytic activity compared to the wt in each condition ± SEM of at least four biological replicates. * p < 0.05, Student’s t-test. **(C)** RP-HPLC analysis of the stem peptides from purified PG isolated from each of the repaired strains. See *Materials and Methods* for a description of the procedures for PG purification, separation of stem peptides, and analysis. Peptides were detected by their absorption at 210 nm and the chemical structures of assigned peaks are diagrammed in S4 Fig.

Given the natural diversity of *murM* alleles among clinical isolates [22,23], we hypothesized that different *murMN* alleles would have different effects on Ply release. To test this, we replaced the Δ*murMN* deletion locus with the *murMN* coding regions from different pneumococcal strains. Thus, each *murMN* allele is under transcriptional control of the native wt *murMN* promoter on the chromosome. Importantly, introduction of the wt *murMN* allele back into Δ*murMN* restored wt levels of Ply release (Fig 4A, *murMN* ^TIGR4^), indicating that the two-fold increase observed upon deletion of *murMN* (Fig 2B) can be attributed specifically to loss of this operon and not due to a second-site mutation that may have occurred elsewhere in the genome during strain construction.

Next, we amplified the *murMN* coding regions from a Pen^S^ (R36A) or Pen^R^ (Pen6) strain [20] and used these to repair Δ*murMN*. Purified MurM derived from a Pen^R^ strain was previously shown to harbor increased catalytic activity *in vitro* compared to a Pen^S^ counterpart [18]. Expression of the *murMN* ^Pen6^ allele caused a two-fold decrease in Ply release as compared to the wt (Fig 4A), representing a four-fold decrease compared to the parent Δ*murMN* strain (compare Fig 2B, *ΔmurMN* to Fig 4A, *murMN* ^Pen6^). This enhanced inhibition of Ply release was specific to *murMN* ^Pen6^, as expression of *murMN* ^R36A^ phenocopied *murMN* ^TIGR4^ (Fig 4A). Thus, restoration of wt levels of Ply release was achieved with either *murMN* ^TIGR4^ or *murMN* ^R36A^, whereas introduction of the highly active *murMN* ^Pen6^ inhibited Ply release to a level comparable to that observed upon *murMN* overexpression in the *malM-murMN* strain.

To address whether these Ply release phenotypes were accompanied by changes in PG stem peptide composition, we purified PG from each repaired strain and analyzed the stem peptide profile as described above. Strikingly, the profiles from each strain expressing a given *murMN* allele were noticeably different than that of the Δ*murMN* parent strain (compare Fig 4C to Δ*murMN* in Fig 2D). Strains expressing *murMN* ^TIGR4^ and *murMN* ^R36A^ exhibited comparable stem peptide profiles to the wt strain with respect to the presence of both linear and branched stem peptides (Fig 4C). While Δ*murMN* lacked any branched monomers (peaks 3 and I), these peptides could be detected in *murMN* ^TIGR4^ and *murMN* ^R36A^ at 3.9% and 4.3%, respectively (Table 1). Additionally, expression of each *murMN* allele caused an approximate two-fold decrease in the directly crosslinked linear dimer (peak 4) compared with Δ*murMN*, accompanied by an increase in the abundance of branched dimers (peaks 5, 6, 7, IV, V, VI) to levels similar to that observed in the wt (Table 1). Thus, the stem peptide profile and Ply release phenotype of Δ*murMN* could be restored by expression of wt *murMN* or the Pen^S^-associated allele from R36A.

Expression of the *murMN* ^Pen6^ allele also lead to the formation of branched stem peptides, albeit to a much greater extent than observed with either of the other two *murMN* alleles tested (Fig 2D). There was significant enrichment in monomers with a branched structure (peaks 3 and I), increasing from zero in *ΔmurMN* to 13.2% (Table 1). Perhaps more striking was the complete reversal in the dimer structures; more than half of the total peptide material of the Δ*murMN* parent was represented by the linear dimer, whereas there was complete replacement of this with the branched forms in *murMN* ^Pen6^ (Table 1). Thus, expression of the Pen^R^-associated *murMN* ^Pen6^ allele caused a shift towards a highly branched PG reminiscent of the *murMN* overexpressing strain. Of note, the stem peptide profile of *murMN* ^Pen6^ is more similar to that of Pen6 itself than the wt strain described herein [20]. Thus, the pneumococcal stem peptide profile is largely dictated by *murMN* expression and activity of the resulting gene products, which is accompanied by differences in Ply release from the cell.

### The incorporation of branched stem peptides is inversely correlated with Ply release

**Fig 5:**
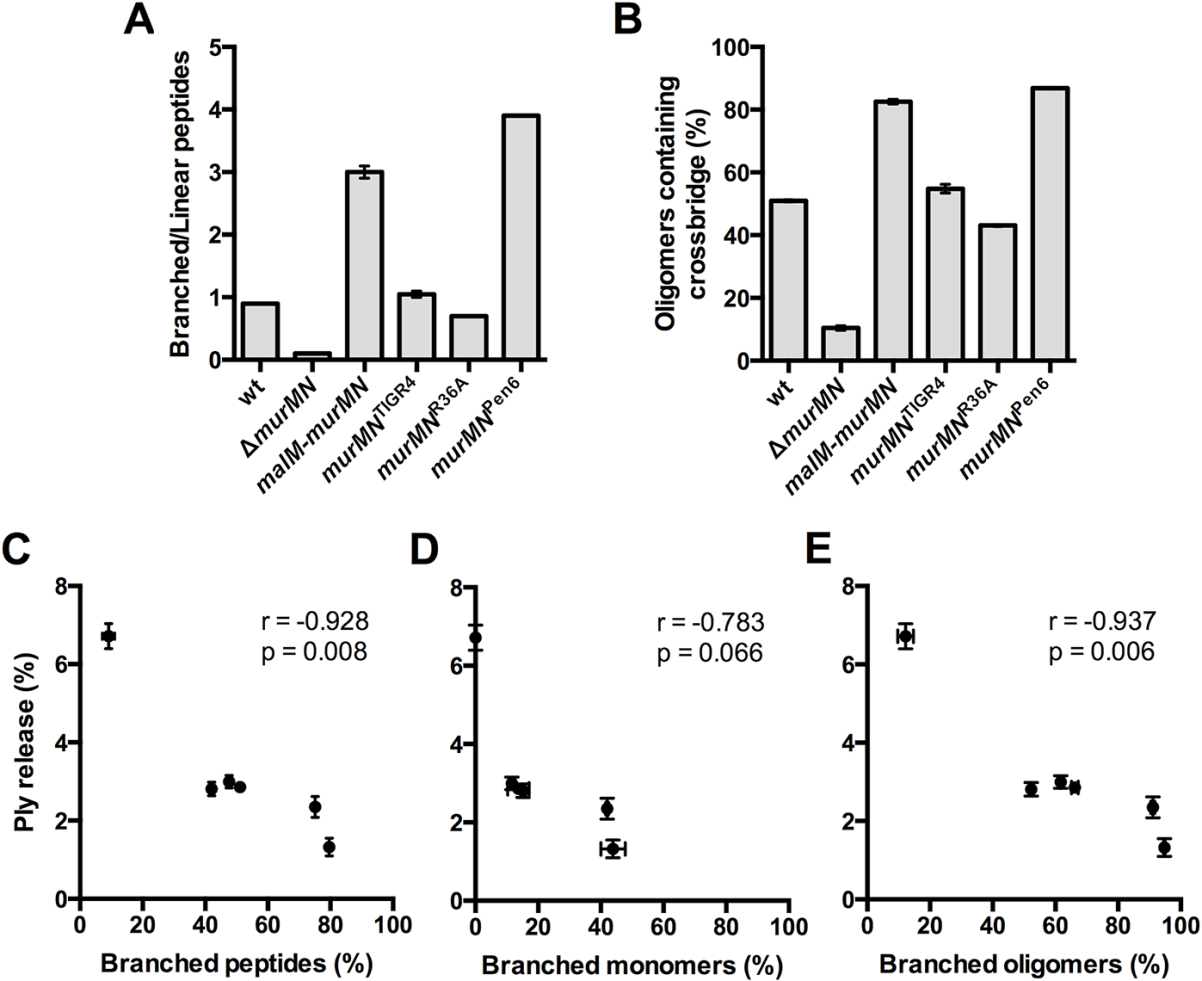
Incorporation of branched stem peptides in PG inversely correlates with Ply release. **(A)** Ratio of branched to linear stem peptides in the PG of indicated strains. **(B)** Percentage of oligomers (dimers and trimers) containing a crossbridge as defined by a crosslink that directly incorporates the branch moiety. **(C-E)** Percent Ply release was calculated using the data from Figs 2B, 2C, 4A and 4B and plotted against the percentage of total branched stem peptides **(C)**, branched monomers **(D)**, and branched oligomers **(E)**. Each symbol represents a given strain. Statistical dependence between percent Ply release and each variable was determined by calculating Pearson’s correlation coefficient (r). In all panels, data are presented as the mean ± SEM.

Stem peptide analysis of the wt and various *murMN* mutants described revealed several differences in discrete peptide species between each strain. We were interested in determining whether there were any global trends from this analysis that could best explain the observed differences in Ply release of each strain. As anticipated, the ratio of branched to linear stem peptides in the total material analyzed was highly dependent on the expression of *murMN*. Deletion of *murMN* caused enrichment in linear stem peptides, whereas *murMN* overexpression led to a three-fold enrichment in branched stem peptides compared to wt (Fig 5A). Repair of Δ*murMN* with the *murMN* ^Pen6^ allele caused a more pronounced enrichment in branched peptides, representing a four-fold increase over the wt (Fig 5A). The incorporation of branched stem peptides into the crosslinked material was also highly dependent on *murMN* and mimicked the trends highlighted in the total peptide material. The percentage of oligomeric species (dimers and trimers) containing a crossbridge, which is indicative of a crosslink that connects the branch structure to an adjacent stem peptide, increased to approximately 80% upon *murMN* overexpression or expression of *murMN* ^Pen6^, which is up from 50% in the wt and the other strains expressing Pen^S^-associated *murMN* alleles (Fig 5B).

Given these gross differences in PG stem peptide profiles we sought to determine whether any specific relationships existed between the PG composition of each strain and the amount of Ply released. We calculated the amount of Ply released as a percentage of the total for each of the strains described in Figs 2 and 4, and plotted it against the total amount of branched stem peptides within each strain. Intriguingly, this analysis revealed a statistically significant, strong negative correlation (Fig 5C). We extended this analysis further by determining whether particular subsets of branched peptide species are more strongly associated with Ply release. There was no significant correlation between Ply release and the percentage of monomers containing a branched structure (Fig 5D). However, the proportion of branched oligomers showed a significant negative correlation with the amount of Ply release observed (Fig 5E). These results suggest that it is not just the presence of branched stem peptides, but also their incorporation into the mature, crosslinked PG that inhibits Ply release.

### Branched stem peptides are required to maintain optimal Ply release during lung infection

**Fig 6:**
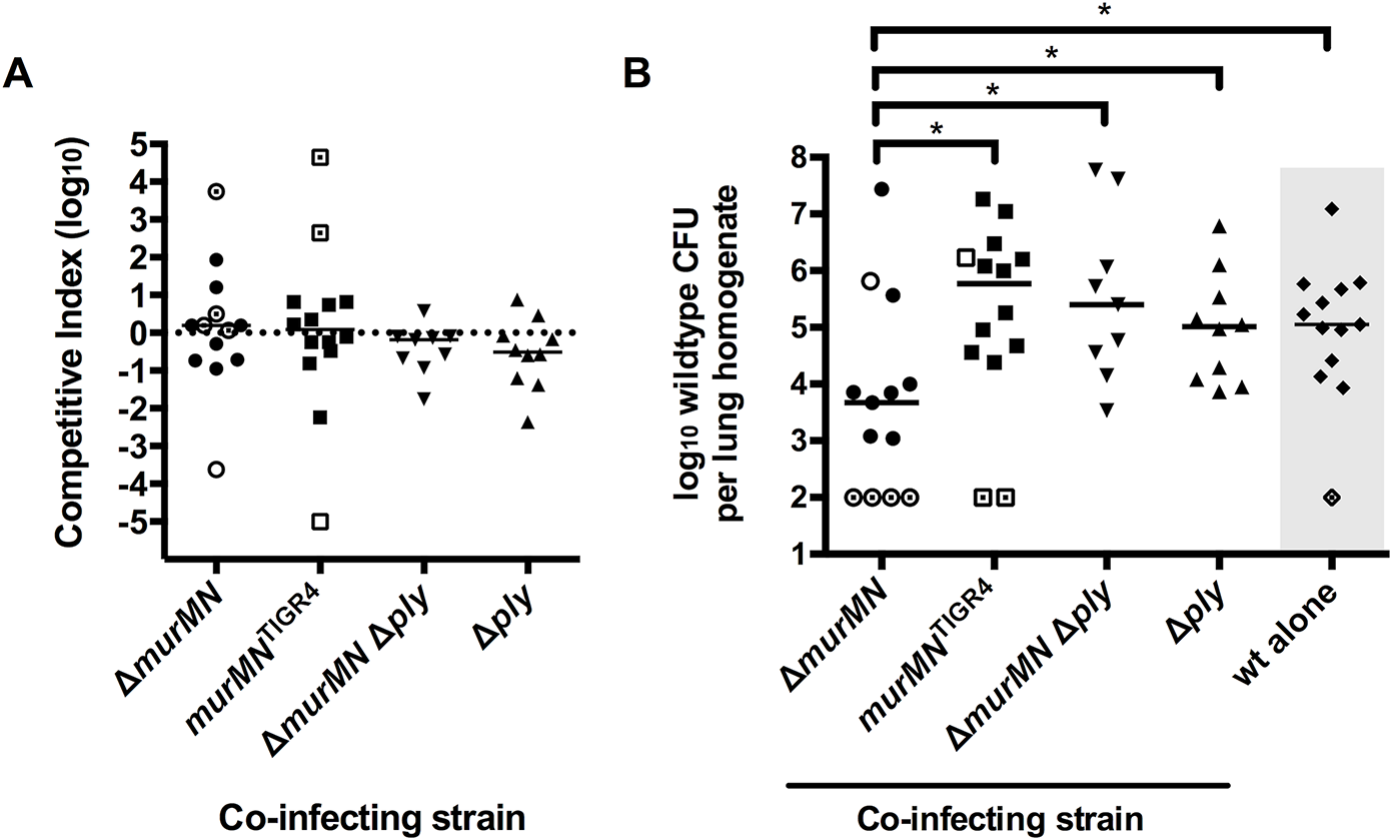
Co-infection with Δ*murMN* decreases wt burdens in a Ply-dependent manner. Equal amounts of the indicated strains were mixed with wt and administered into the lungs of mice via intranasal inoculation. A competitive index (CI) **(A)** and the recovered wt titers **(B)** were determined for each mouse in each sample group. As a control, single-strain infections with wt alone were performed and the titers are depicted in panel B (indicated by the gray box). Each symbol represents an individual mouse and the bars indicate the median CI **(A)** or median bacterial titer **(B)**. Open symbols without or with a central dot indicate titers that are at or below the limit of detection of 100 CFU/homogenate for the mutant or wt, respectively. The median bar masks two open, central dot symbols in the Δ*murMN* dataset. No group in **(A)** has a median CI that is significantly different from 1.0 as determined by Wilcoxon Signed Rank test. * p<0.05; Mann-Whitney test.

To assess the contribution of branched stem peptides and Ply release to pneumococcal virulence, we competed Δ*murMN* or *murMN* ^TIGR4^ against wt in a murine model of pneumonia. Neither mutant demonstrated a fitness defect as determined by the competitive index (Fig 6A). However, infection with Δ*murMN* caused a 125-fold decrease in the median number of recovered wt bacteria as compared to the *murMN* ^TIGR4^ competition (Fig 6B). As a control, we also performed single-strain lung infections with wt and found that the titers achieved by the wt alone were 23-fold higher than those during co-infection with Δ*murMN* (Fig 6B). However, wt reached similar titers either alone or during competition with the repaired *murMN* ^TIGR4^ strain (Fig 6B, compare wt alone to *murMN* ^TIGR4^, medians not significantly different), suggesting that the absence of branched stem peptides in Δ*murMN* negatively impacts virulence.

We hypothesized that the increased Ply release in the Δ*murMN* strain (Fig 2B) was detrimental to the virulence of the co-infecting wt strain. To address whether the inhibitory effect of co-infection with Δ*murMN* was Ply-dependent, we deleted the *ply* structural gene in the Δ*murMN* background and competed this double mutant against wt *in vivo*. In support of our hypothesis, wt titers increased 53-fold compared to the Δ*murMN* co-infection and reached levels that were statistically indistinguishable from those observed during competition with *murMN* ^TIGR4^ (Fig 6B, compare Δ*murMN* Δ*ply* to *murMN* ^TIGR4^, medians not significantly different). As a control, we also competed the single Δ*ply* mutant against wt and were able to recover wt bacteria to approximately the same level as in co-infection with *murMN* ^TIGR4^ or Δ*murMN* Δ*ply* (Fig 6B). Similar to the other strains tested, neither Δ*murMN* Δ*ply* nor Δ*ply* showed a fitness defect during competition with the wt (Fig 6A). Taken together, co-infection with Δ*murMN* limits the ability of wt to achieve high titers *in vivo* and this inhibitory effect can be relieved by restoration of the *murMN* operon or deletion of *ply*. This strongly suggests that appropriate Ply release *in vivo* is dependent on PG stem peptide composition and maintenance of this ability is necessary for pneumococcal virulence.

## Discussion

Despite the lack of a signal peptide and a cell surface-localization motif, Ply appears to be exported through the pneumococcal membrane and associates with the cell envelope by an unresolved mechanism [12,13]. In this study, we demonstrate that exported Ply is not surface-accessible and its activity and release into the surrounding medium is dependent on enzymatic digestion of the PG layer. This may suggest that surface-localized Ply is inhibited due to an inability to fold into a pore-forming competent state while associated with the cell envelope. Alternatively, but not mutually exclusive, the accumulation of Ply at this site may be due to restricted mobility of the exported protein through the PG matrix. Our results cannot distinguish between these two possibilities but it is clear from our experiments that Ply activity and release is dependent on PG hydrolysis.

Theoretical and experimental analysis has suggested that purified PG can accommodate globular proteins ranging from ~25–50 kilodaltons (kDa) [34]. However, this likely represents a static portrait of PG pore size, as it does not account for the dynamic remodeling of this layer that occurs during cell growth [14,35]. Ply is a 53-kDa protein, putting it on the upper boundary of the experimentally defined range noted above. The existence of a “periplasm-like” space delineated by the plasma membrane and cell wall matrix within the Gram-positive cell envelope has been proposed by several groups and experimentally investigated in different species [36–39]. It is tempting to speculate that, upon export through the membrane, Ply is transiently restricted within this “periplasm-like” space due to its limited ability to diffuse through the cell wall matrix, which may be modulated by altering the proportion of branched stem peptides in PG.

Interestingly, Ply was released from washed pneumococci in the absence of host cells (i.e. red blood cells), suggesting that this process may not strictly require a host-derived signal or direct contact. This is in contrast to the pH-dependent release of phospholipase C (PC-PLC) from the cell wall of the intracellular pathogen *Listeria monocytogenes* [40]. PC-PLC, like Ply, lacks a sorting motif yet localizes to the cell wall compartment of *L*. *monocytogenes* and, under native conditions, exists in a state that cannot be detected with antibodies; though digestion of the PG layer allows for complete release and exposure [40,41]. Entry into the host cell cytosol causes a rapid release of surface-associated PC-PLC only upon acidification of the intracellular environment, which is required for optimal virulence of *L*. *monocytogenes* [40]. Attempts to detect surface-localized Ply by immunofluorescence have been unsuccessful, perhaps due to the inability of antibodies to reach the niche that Ply occupies within the cell envelope. It is important to note that we cannot rule out the possibility that a specific trigger exists *in vivo* to stimulate Ply release, however, it appears that this stimulus is not required *per se* and that release is at least partly dependent on PG structure and the presence of CBPs on the cell surface. Of note, a recent report demonstrated that phagocytosis of the pneumococcus by macrophages results in Ply-dependent death of both host cell and bacterium. This phenomenon was potentiated in a mutant exhibiting hypersensitivity to lysozyme digestion, thus implicating this host factor in contributing to Ply release *in vivo* [42].

Pneumococcal PG contains both linear and branched stem peptides, the proportions of which vary depending on the expression level of *murMN* as well as the activity of the encoded proteins (Figs 2D, 4C and Table 1) [17,29]. Branched stem peptide formation was inversely related to the amount of Ply released as determined through deletion and overexpression of the *murMN* operon. MurM and MurN are non-essential and the pneumococcus grows similarly *in vitro* both with and without branched stem peptides [20]. Therefore, the precise role of branched stem peptides in pneumococcal biology remains an open question. Previous observations demonstrated that *murMN* expression was associated with an altered propensity to autolyse upon treatment with a variety of cell wall-targeting compounds suggesting that differences in stem peptide composition confer altered susceptibility to autolysis [24]. However, the underlying mechanisms of this phenomenon are unclear.

One clue that may help illuminate this issue comes from our studies of Ply and LacZ release from the *murMN* deletion and overexpression mutants. Our observations suggest that PG lacking branched stem peptides (exemplified by Δ*murMN*) may be more sensitive to the action of a subset of CBPs capable of mediating PG hydrolysis even in the absence of any overt perturbation to the cell wall and/or membrane. This sensitivity can be suppressed by increasing the abundance of branched stem peptides within the PG layer. Whether variation in branched stem peptide abundance is accompanied by altered susceptibility of the PG layer to the action of PG hydrolases, including those that are CBPs, remains an interesting and unexplored possibility. Furthermore, these results suggest that Ply can be released specifically from the cell surface in the absence of lysis, which is likely the result of controlled PG remodeling by CBPs. Intriguingly, a recent report revealed a role for PG hydrolases (including pneumococcal LytA, a CBP) in limiting recognition by peptidoglycan responsive proteins of the innate immune system through controlled trimming of the PG layer [43]. This supports the hypothesis that regulated PG cleavage is critical during infection for additional reasons other than promoting cell growth and division.

Removal of CBPs from the cell surface caused an overall decrease in both Ply and LacZ release, especially in the Δ*murMN* mutant (Fig 3H). However, both proteins could be detected in equal proportions, suggesting cytoplasmic leakage due to autolysis that may be the result of incomplete removal of surface-bound CBPs or the activity of other, non-choline-binding PG hydrolases. Of note, the pneumococcus encodes numerous known and putative PG hydrolases that are likely unaffected by choline treatment [44]. Intriguingly, CBP-mediated autolysis was also recently shown to be necessary for the release of LytA amidase from the cytoplasm during logarithmic growth [45]. LytA, like Ply, lacks a N-terminal signal peptide suggesting that basal autolysis during growth can allow for the release of other leaderless extracellular proteins that may then reassociate with the cell surface. However, it is important to point out that Ply released upon autolysis was not able to bind to the surface of actively growing cells [13].

The association between Pen^R^ and *murMN* led us to test whether Ply release would be affected by the expression of altered *murMN* alleles that appear to naturally arise in response to antibiotic pressure. Repair of the Δ*murMN* mutant with a Pen^R^-associated *murMN* allele, but not a Pen^S^-associated allele, caused a four-fold reduction in the amount of Ply release compared to the Δ*murMN* parent strain accompanied by significant changes to the stem peptide profile. Given that each allele was under control of the endogenous *murMN* promoter on the chromosome, these data support previous observations that variant Pen^R^-associated *murM* alleles encode proteins with higher catalytic activity compared to a Pen^S^ MurM [18]. Also, this lends support to the model that PG containing more branched stem peptides represents a more restrictive environment for Ply release.

The biological consequences of Pen^R^ can lead to pleiotropic effects that can cause fitness defects in different pathogens, including the pneumococcus [46–48]. The precise mechanism(s) that select for *murMN* mutations in the context of Pen^R^ low-affinity PBPs are not well understood. There is some evidence suggesting that certain PBPs display different substrate specificities for transpeptidation [49,50]. Given that low-affinity PBPs demonstrate decreased affinity for penicillin, which is structurally similar to their natural substrate, it seems plausible to speculate that selection for low-affinity PBPs could create a PG structure that may phenocopy the loss of *murMN* with respect to both Ply release and decreased virulence. Therefore, this fitness defect may aid in selection for *murMN* mutations that compensate for altered PG architecture and Ply release. Whether Ply release plays a role in the selection for compensatory mutations in the context of low-affinity PBPs is an intriguing hypothesis that remains to be explored.

Infection with the *murMN* deletion mutant caused a significant Ply-dependent decrease in the ability of wt to achieve high titers *in vivo*. Perhaps more surprising was that, despite the gross changes in stem peptide composition, this mutant was as fit as the wt during competitive lung infection. Importantly, this result indicates that the altered stem peptide composition in the Δ*murMN* mutant does not result in a fitness defect in this strain compared to the wt. Ply is a potent inflammatory agonist that contributes to cytokine production and can cause influx of phagocytic cells [9,51]. Therefore, we propose that the increased Ply release caused by infection with Δ*murMN* causes a robust, perhaps premature, immune response that results in enhanced bacterial clearance. Neither the wt nor the mutant is equipped to handle this heightened response, which results in a reduction of both strains during this co-infection. It is unclear whether the pneumococcus modulates the proportion of branched stem peptides in the PG layer during infection. Of note, *murM* expression appears to be specifically upregulated in the presence of epithelial cells *in vitro* [52], supporting the hypothesis that the stem peptide composition of PG may be actively modulated under certain conditions mimicking those found *in vivo*.

The finding that repair of Δ*murMN* to a *murMN* ^*+*^ state restored both Ply release *in vitro* and caused wt titers to rebound *in vivo* strongly suggests that it is the specific localization of Ply, not the amount produced, that dictates successful infection. Both of these strains are *ply* ^+^ and produce the same amount of total Ply *in vitro*. Thus, the discrepancy in bacterial loads *in vivo* observed between the groups appears to be independent of Ply production. Indeed, a Δ*ply* mutant did not exhibit a fitness defect during co-infection in this model (Fig 6A). At first glance, this appears to be in opposition to the dogma that Ply is a critically important virulence factor of the pneumococcus [53–55]. However, these classical studies were all conducted using single-strain infections with either wt or a *ply* mutant and not a competition between the two. Indeed, our results are consistent with the observations of Benton *et al*, who demonstrated that growth of a *ply* mutant in a blood model of infection could be rescued upon co-infection with a wildtype strain [56]. The lack of attenuation observed for Δ*ply* during competition against the wt actually provides further support for our argument that Ply is released from the cell wall compartment during infection as it appears the Δ*ply* strain can be complemented in *trans* by Ply released from the wt. Based on this notion, we propose that the dynamics of Ply release in wt cells is carefully balanced so as to promote virulence without causing immune-mediated clearance.

In summary, we have demonstrated that Ply release from the pneumococcal cell wall compartment and cytoplasm can occur in the absence of a host signal but is limited by the native PG network. Based on these observations, we propose that PG acts as a barrier that causes an accumulation of exported Ply at the cell surface that may be released upon remodeling by PG hydrolases, particularly those that are CBPs. Additionally, we identified a novel role for branched stem peptides in restricting Ply release from the cell presumably by affecting the type of crosslinks that create the PG structure. The acquisition of Pen^R^, and other determinants necessary for its expression, may differentially affect Ply release and, as a result, the outcome of an infection. Finally, we demonstrate that branched stem peptides play a critical role in maintaining the PG barrier, which allows for precise control over Ply release. We propose that this is necessary to establish the balance between virulence and immune activation, thus suggesting that PG architecture may play an important regulatory role in the pathogenesis of diverse microbial pathogens. This is the first indication that the cell wall-associated Ply may contribute to pneumococcal virulence.

## Materials and Methods

### Bacterial strains and growth conditions

All experiments were performed with the serotype 4 strain, TIGR4, or its isogenic mutant derivatives, which are described in Table 2. Strains were routinely grown from frozen glycerol stocks on tryptic soy agar plates containing 5% sheep blood (Northeast Laboratory) overnight at 37°C in a 5% CO_2_ environment. The growth from overnight plates was suspended in Todd Hewitt broth (BD Biosciences) supplemented with 0.5% (wt/vol) yeast extract (Fisher Scientific) (THY medium), diluted to an OD_600_ of 0.02, and grown to a final OD_600_ of 0.8 in a 37°C incubator with 5% CO_2_ for all experiments. THY medium was routinely supplemented with 0.5% Oxyrase (Oxyrase, Inc.). When necessary, the following antibiotics at the indicated concentrations were used: chloramphenicol (Cm) (4 μg/mL) and spectinomycin (Spec) (200 μg/mL).

**Table 2:** Strains and plasmids used in the present study.

Strain collection ID	Strain name	Genotype and antibiotic resistance(s)	Description	Source
***Streptococcus pneumoniae***				
AC316	wt	Wildtype	TIGR4 (serotype 4)	Laboratory strain
AC4382	Δ*murMN*	Δ*murMN*::cat; Cm^R^	Replacement of the *murMN* (SP_0615-SP_0616) operon with cat cassette	This work
AC4404	*malM-murMN*	*malM-murMN-*cat; Cm^R^	Transcriptional fusion of *murMN* and cat cassette to *malM* (SP_2107)	This work
AC4108	Δ*ply*	Δ*ply*::cat; Cm^R^	Replacement of *ply* (SP_1923) with cat cassette	[60]
AC4453	Δ*murMN* Δ*ply*	Δ*murMN*::cat, Δ*ply*::spec; Cm^R^, Spec^R^	Replacement of *ply* with spec cassette in the AC4382 background	This work
AC4498	*murMN* ^TIGR4^	Δ*murMN*::*murMN* ^TIGR4^-spec; Spec^R^	Replacement of cat cassette in AC4382 with *murMN* amplified from strain TIGR4 fused to spec cassette	This work
AC4499	*murMN* ^R36A^	Δ*murMN*::*murMN* ^R63A^-spec; Spec^R^	Replacement of cat cassette in AC4382 with *murMN* amplified from strain R36A fused to spec cassette	This work, [61]
AC4500	*murMN* ^Pen6^	Δ*murMN*::*murMN* ^Pen6^-spec; Spec^R^	Replacement of cat cassette in AC4382 with *murMN* amplified from strain Pen6 fused to spec cassette	This work, [62]
AC5073	wt *lacZ*	Δ*bgaA*::spec-*lacZ*; Spec^R^	Replacement of *bgaA* (SP_0648) coding region in AC316 with *E*. *coli lacZ* transcriptionally fused to spec cassette	This work
AC5074	Δ*murMN lacZ*	Δ*murMN*::cat, Δ*bgaA*::spec-*lacZ*; Cm^R^, Spec^R^	Replacement of *bgaA* (SP_0648) coding region in AC4382 with *E*. *coli lacZ* transcriptionally fused to spec cassette	This work
AC5075	*malM-murMN lacZ*	*malM-murMN*-cat, Δ*bgaA*::spec-*lacZ*; Cm^R^, Spec^R^	Replacement of *bgaA* (SP_0648) coding region in AC4404 with *E*. *coli lacZ* transcriptionally fused to spec cassette	This work
***Escherichia coli***				
AC578	DH5α λpir pAC578	pAC578; Spec^R^	Source of spectinomycin resistance (spec) cassette	[63]
AC1000	DH5α pAC1000	pAC1000; Cm^R^	Source of chloramphenicol resistance (cat) cassette	[30]
AC3450	MG1655	Wildtype strain	Source of *lacZ*	Laboratory strain

### Mutant strain construction

Mutations were generated using allelic exchange with linear PCR amplicons. For deletion constructs, linear amplicons were created via splicing by overlap extension (SOE) PCR. 1 kb of flanking sequence immediately upstream and downstream of the target gene was amplified from TIGR4 gDNA and fused to either a Cm^R^ or Spec^R^ cassette, which were amplified from pAC1000 and pAC578 (Table 2), respectively. To repair Δ*murMN* with different *murMN* alleles, the coding regions and intervening sequence of *murMN* were amplified from gDNA prepared from the appropriate strains (Table 2) and fused to a Spec^R^ cassette and the flanking arms of homology surrounding the native *murMN* locus. Construction of the *murMN* overexpression strain was done essentially as described [30]. Generation of *lacZ-*expressing strains was performed by replacing the coding region of *bgaA* (SP_0648) on the pneumococcal chromosome with a transcriptional fusion of *E*. *coli lacZ* to the Spec^R^ cassette. To do so, the coding region of *lacZ* amplified from *E*. *coli* MG1655 gDNA (Table 2) was placed downstream of the Spec^R^ cassette and was flanked on either side by 1 kb of sequence immediately upstream and downstream of the *bgaA* coding region by SOE PCR. Transformation of the pneumococcus was carried out as described previously [57]. All mutant constructs were verified using PCR and DNA sequencing.

### Hemolysis assays

Mid-exponential growth phase cells were normalized to OD_600_ = 0.8 (approx. 10^8^ CFU/mL), collected by centrifugation, washed once in assay buffer [AB; phosphate-buffered saline (PBS), 0.1% bovine serum albumin, 10 mM dithiothreitol] and resuspended in AB. An aliquot of each sample was sonicated at 4°C at maximum amplitude for 2 minutes in a water bath sonicator (Branson, Inc.) using a 10 second on, 5 second off duty cycle. Whole cell, sonicated, or subcellular fractions were serially 2-fold diluted in AB in 96-well V-bottom plates (Greiner Bio-One). For each experiment, an 8% solution of triple-washed sheep red blood cells (SRBC) was freshly prepared and 50 μL of this was added to each well containing 100 μL of sample or control wells containing either AB alone or distilled water. To test whether the cell-free supernatant harbored hemolytic activity, AB alone was substituted for the SRBC solution in the first hour incubation and then 100 μL of the supernatant was transferred to a new plate to which SRBC was added as described above. Plates were incubated at 37°C for 1 hour after which the bacterial cells and any unlysed SRBC were pelleted by centrifugation at 4000 x g for 10 min. One hundred microliters of each supernatant was removed to a 96-well flat bottom plate and the absorbance at 570 nm was measured. After subtracting the AB only value from each sample, the percent activity from each well was determined relative to the distilled water control, which was set to 100% hemolysis. Plotting the OD_570_ versus the OD_600_ of each sample yielded a sigmoidal curve, from which the linear portion of the curve was used to extrapolate the OD_600_ at which 50% hemolysis occurred. The reciprocal of this value is defined as the number of hemolytic units and each whole cell sample or subcellular fractionation was divided by its paired sonicated sample to determine percent hemolytic activity.

For removal of choline-binding proteins, strains were grown and processed for hemolysis assays as described above with the following modifications. After washing away media, cells were washed once in PBS and split evenly in two separate tubes. After centrifugation, pellets were concentrated five-fold in either PBS or PBS containing 2% choline chloride (Sigma) and incubated at room temperature with agitation for 20 minutes. After centrifugation and washing once in AB, an aliquot was removed for sonication to determine total hemolytic activity and the remainder of each sample was incubated in AB at 37°C for 1 hour. Cells were collected by centrifugation and the supernatant, containing released proteins, was assayed for hemolytic activity as described above.

### Miller assays

Released and sonicated fractions were prepared as described in the section above and the presence of LacZ in each sample was determined by β-galactosidase assays using the colorimetric substrate ortho-nitrophenyl-β-D-galactopyranoside (ONPG) (Sigma) as described in [58]. A control reaction containing ONPG alone was included in all experiments and served as the blank sample. Preliminary experiments demonstrated that whole cell lysates from a strain lacking *bgaA* (endogenous β-galactosidase) produced background levels of ONPG hydrolysis. Serial five-fold dilutions of Δ*bgaA*::*spec-lacZ* (AC5073) whole cell lysates in triplicate revealed that β-galactosidase activity was linear down to 0.89±0.014% of the starting material (OD_600_ = 0.8) (S5 Fig).

### Peptidoglycan purification and enzymatic digestion

Cell wall material was prepared using a previously published protocol [20]. Peptidoglycan was further purified from approximately 5–20 mg of cell wall material by treatment with 48% hydrofluoric acid for 48 hours at 4°C with agitation. Hydrofluoric acid was removed through extensive washing with 100 mM Tris-HCl pH 7.0 and its absence confirmed by measuring the pH. Purified peptidoglycan was then collected by lyophilization and subjected to enzymatic digestion with purified pneumococcal LytA amidase to liberate stem peptides as described previously [20].

### Analysis of stem peptide composition

Stem peptides were separated and analyzed by reversed phase-high performance liquid chromatography (RP-HPLC) as described elsewhere [20].

### Subcellular fractionation

Cell wall digestion was performed as previously described [13], with some modifications. Briefly, cells from exponentially growing cultures were collected by centrifugation, washed once with 50 mM Tris-Cl, pH 7.5 and resuspended in 100 μL cell wall digestion buffer [50 mM Tris-Cl, pH 7.5, 30% (w/v) sucrose, 1 mg/mL lysozyme, 300 U/μL mutanolysin, 1x protease inhibitor cocktail (Roche)]. Cell wall digestion was allowed to proceed for 2 hours at 37°C on a roller drum after which protoplasts were separated from cell wall material via centrifugation at 13,000 x g for 10 min. The supernatant was collected as the cell wall fraction. Protoplasts were resuspended in 100 μL 50 mM Tris-Cl, pH 7.5.

### Western blot analysis

An equivalent amount of each subcellular fraction was mixed with 2x Laemmli sample buffer and heated in a boiling water bath for 10 minutes prior to loading cell equivalents on a 10% SDS-PAGE gel. Gels were run at 125 V until the dye front reached the bottom of the gel and then proteins were transferred to a nitrocellulose membrane at 25 V for 1.5 hours. Membranes were blocked with NAP-blocker (G-Biosciences) diluted 1:2 with Tris-buffered saline containing 0.1% Tween-20 (TBST) and then cut to allow for simultaneous probing with each antibody. Primary antibodies against Ply at 1:1000 (Statens Serum Institut) and CodY at 1:1500 (a gift from A.L. Sonenshein) were diluted accordingly in NAP-blocker mixed 1:4 with TBST and applied to each membrane at room temperature for 1 hour with rocking. Membranes were washed three times for 5 minutes each with TBST. Appropriate Cy5-conjugated secondary antibody at 1:1000 (Invitrogen, Inc.) was applied to each membrane as described above for the primary and then each blot was washed as described above. Membranes were scanned with a Fuji FLA-9000 instrument and the amount of fluorescence was quantitated using MultiGauge analysis software (Fujifilm, Corp.)

### Peptidoglycan binding assay

Wildtype cells from a mid-log culture were concentrated to an OD_600_ of 3.5 in PBS and sonicated as described above. After centrifugation to remove cellular debris, the supernatant was transferred to a new tube and used as the source of Ply. Pull-down assays were performed to determine if Ply binds PG. To this end, purified wt PG was mixed with wt cell lysate in a final reaction volume of 30 μL and allowed to incubate on a roller drum at 37°C for 2 hours. Control samples lacking either PG or lysate were performed in parallel. Insoluble PG was pelleted by centrifugation at 20,000 x *g* for 30 minutes at room temperature and the supernatant (unbound fraction) was removed to a separate tube. The pellet (bound fraction) was washed once with 90 μL PBS, collected by centrifugation, and then resuspended in 30 μL PBS. The presence of Ply within each supernatant and pellet fraction was detected by Western blot analysis as described above with the exception that samples were resolved by SDS-PAGE using NuPAGE 4–12% Bis-Tris gels (Life Technologies) run at 200 V for 35 minutes.

### qRT-PCR

RNA extraction and qRT-PCR was performed exactly as described [59] to analyze *murM* and *murN* transcript levels in exponentially growing cultures of wt and *malM-murMN* grown in THY medium with and without added 0.8% maltose.

### Animal infections

Lung infections were carried out as described in [59] except that mice were euthanized 30–36 hours post-inoculation.

### Ethics statement

All animal experiments were done in accordance with NIH guidelines, the Animal Welfare Act and US federal law. Tufts University School of Medicine’s Institutional Animal Care and Use Committee approved the experimental protocol “B2014-37” that was used for this study. All animal experiments were housed in a centralized and AAALAC-accredited research animal facility that is fully staffed with trained husbandry, technical and veterinary personnel.

## Supporting Information

S1 Fig Ply localization to the cell wall compartment is unaffected by mutations in the murMN operon. **(A)** The indicated strains were subjected to subcellular fractionation to separate cell wall (CW) material from protoplasts (Prt) and the amount of Ply and CodY present in each fraction was determined by Western blot analysis. A representative image of two independent experiments is shown. **(B)** The amount of Ply present in the cell wall fraction relative to the total amount of Ply (cell wall and protoplast) normalized to the cytoplasmic protein CodY for each of the strains analyzed was quantitated for two independent experiments and is shown relative to wt for each condition tested.
(TIFF)

S2 Fig Ply does not bind PG in a pull-down assay. **(A)** A wt cell lysate was mixed with different amounts of purified wt PG (2.6 mg/mL to 0.17 mg/mL, 2-fold serial dilutions). **(B)** A fixed amount of purified wt PG (1.3 mg/mL) was mixed with different amounts of wt cell lysate (OD_600_ = 3.5 to 0.2 of initial culture, 2-fold serial dilutions). PG alone (1.3 mg/mL) or lysate alone (no PG) were included as controls. Samples were incubated at 37°C on a roller drum for 2 hours. Insoluble PG was pelleted by centrifugation at 21,000 x *g* for 30 minutes. The supernatant (S) containing unbound material was removed and the pellet (P) representing the bound fraction was washed once with PBS and resuspended in an equal volume to the supernatant to normalize cell equivalents. The amount of Ply present in each sample was determined by Western blot analysis. In each panel, a representative image of two independent experiments is shown.
(TIFF)

S3 Fig Quantification of murMN expression in the malM-murMN strain grown with and without inducer. Relative expression of *murM* and *murN* compared to wt (set to 1) in *malM-murMN* grown in the absence (THY) or presence (THY + 0.8% maltose) of inducer. Transcript abundance was normalized to the housekeeping gene, *rplI*.
(TIFF)

S4 Fig Peptide structures of the indicated peaks in Figs 2D and 4C. (TIFF)

S5 Fig Sensitivity of the β-galactosidase assay used to measure LacZ release. Three independent cultures of Δ*bga*::*spec-lacZ* (AC5073) were grown to OD_600_ = 0.8, pelleted by centrifugation, resuspended in hemolysis assay buffer, and sonicated to generate whole cell lysates as described in *Materials and Methods*. Each lysate was serially five-fold diluted three times to make a dilution series (1x, 0.2x, 0.04x, 0.008x). **(A)** β-galactosidase activity of each dilution was detected using Miller assays. **(B)** Miller units of each dilution plotted as a function of the percent starting material yields a linear relationship down to 0.89%. Each symbol represents three overlapping symbols that each depicts a biological replicate. The coefficient of determination (R^2^) was determined using GraphPad Prism 6 software.
(TIFF)

## References

[1] Bogaert D, de Groot R, Hermans PWM (2004) *Streptococcus pneumoniae* colonisation: the key to pneumococcal disease. Lancet Infect Dis 4: 144–154. 10.1016/S1473-3099(04)00938-7 14998500

[2] Kadioglu A, Weiser JN, Paton JC, Andrew PW (2008) The role of *Streptococcus pneumoniae* virulence factors in host respiratory colonization and disease. Nat Rev Microbiol 6: 288–301. 10.1038/nrmicro1871 18340341

[3] Rubins JB, Duane PG, Charboneau D, Janoff EN (1992) Toxicity of pneumolysin to pulmonary endothelial cells in vitro. Infect Immun 60: 1740–1746. 1563759 10.1128/iai.60.5.1740-1746.1992 PMC257067

[4] Zysk G, Schneider-Wald BK, Hwang JH, Bejo L, Kim KS, et al (2001) Pneumolysin is the main inducer of cytotoxicity to brain microvascular endothelial cells caused by *Streptococcus pneumoniae* . Infect Immun 69: 845–852. 10.1128/IAI.69.2.845–852.2001 11159977 PMC97961

[5] Baba H, Kawamura I, Kohda C, Nomura T, Ito Y, et al (2001) Essential role of domain 4 of pneumolysin from *Streptococcus pneumoniae* in cytolytic activity as determined by truncated proteins. Biochem Biophys Res Commun 281: 37–44. 10.1006/bbrc.2001.4297 11178957

[6] Paton JC, Rowan-Kelly B, Ferrante A (1984) Activation of human complement by the pneumococcal toxin pneumolysin. Infect Immun 43: 1085–1087. 6698602 10.1128/iai.43.3.1085-1087.1984 PMC264298

[7] Ratner AJ, Hippe KR, Aguilar JL, Bender MH, Nelson AL, et al (2006) Epithelial cells are sensitive detectors of bacterial pore-forming toxins. J Biol Chem 281: 12994–12998. 10.1074/jbc.M511431200 16520379 PMC1586115

[8] Shoma S, Tsuchiya K, Kawamura I, Nomura T, Hara H, et al (2008) Critical involvement of pneumolysin in production of interleukin-1α and caspase-1-dependent cytokines in infection with *Streptococcus pneumoniae* in vitro: a novel function of pneumolysin in caspase-1 activation. Infect Immun 76: 1547–1557. 10.1128/IAI.01269-07 18195026 PMC2292879

[9] McNeela EA, Burke Á, Neill DR, Baxter C, Fernandes VE, et al (2010) Pneumolysin activates the NLRP3 inflammasome and promotes proinflammatory cytokines independently of TLR4. PLoS Pathog 6: e1001191 10.1371/journal.ppat.1001191 21085613 PMC2978728

[10] Walker JA, Allen RL, Falmagne P, Johnson MK, Boulnois GJ (1987) Molecular cloning, characterization, and complete nucleotide sequence of the gene for pneumolysin, the sulfhydryl-activated toxin of *Streptococcus pneumoniae* . Infect Immun 55: 1184–1189. 3552992 10.1128/iai.55.5.1184-1189.1987 PMC260488

[11] Balachandran P, Hollingshead SK, Paton JC, Briles DE (2001) The autolytic enzyme LytA of *Streptococcus pneumoniae* is not responsible for releasing pneumolysin. J Bacteriol 183: 3108–3116. 10.1128/JB.183.10.3108–3116.2001 11325939 PMC95211

[12] Price KE, Camilli A (2009) Pneumolysin localizes to the cell wall of *Streptococcus pneumoniae* . J Bacteriol 191: 2163–2168. 10.1128/JB.01489-08 19168620 PMC2655535

[13] Price KE, Greene NG, Camilli A (2012) Export requirements of pneumolysin in *Streptococcus pneumoniae* . J Bacteriol 194: 3651–3660. 10.1128/JB.00114-12 22563048 PMC3393478

[14] Typas A, Banzhaf M, Gross CA, Vollmer W (2011) From the regulation of peptidoglycan synthesis to bacterial growth and morphology. Nat Rev Microbiol 10: 123–136. 10.1038/nrmicro2677 22203377 PMC5433867

[15] Schleifer KH, Kandler O (1972) Peptidoglycan types of bacterial cell walls and their taxonomic implications. Bacteriol Rev 36: 407–477. 4568761 10.1128/br.36.4.407-477.1972 PMC408328

[16] Garcia-Bustos JF, Chait BT, Tomasz A (1987) Structure of the peptide network of pneumococcal peptidoglycan. J Biol Chem 262: 15400–15405. 2890629

[17] Filipe SR, Pinho MG, Tomasz A (2000) Characterization of the *murMN* operon involved in the synthesis of branched peptidoglycan peptides in *Streptococcus pneumoniae* . J Biol Chem 275: 27768–27774. 10.1074/jbc.M004675200 10869361

[18] Lloyd AJ, Gilbey AM, Blewett AM, De Pascale G, Zoeiby El A, et al (2008) Characterization of tRNA-dependent peptide bond formation by MurM in the synthesis of *Streptococcus pneumoniae* peptidoglycan. J Biol Chem 283: 6402–6417. 10.1074/jbc.M708105200 18077448

[19] De Pascale G, Lloyd AJ, Schouten JA, Gilbey AM, Roper DI, et al (2008) Kinetic characterization of lipid II-Ala:alanyl-tRNA ligase (MurN) from *Streptococcus pneumoniae* using semisynthetic aminoacyl-lipid II substrates. J Biol Chem 283: 34571–34579. 10.1074/jbc.M805807200 18842590 PMC3259876

[20] Filipe SR, Tomasz A (2000) Inhibition of the expression of penicillin resistance in *Streptococcus pneumoniae* by inactivation of cell wall muropeptide branching genes. Proc Natl Acad Sci USA 97: 4891–4896. 10.1073/pnas.080067697 10759563 PMC18328

[21] Garcia-Bustos J, Tomasz A (1990) A biological price of antibiotic resistance: major changes in the peptidoglycan structure of penicillin-resistant pneumococci. Proc Natl Acad Sci USA 87: 5415–5419. 2371278 10.1073/pnas.87.14.5415 PMC54335

[22] Smith AM, Klugman KP (2001) Alterations in MurM, a cell wall muropeptide branching enzyme, increase high-level penicillin and cephalosporin resistance in *Streptococcus pneumoniae* . Antimicrob Agents Chemother 45: 2393–2396. 10.1128/AAC.45.8.2393–2396.2001 11451707 PMC90664

[23] Filipe SR, Severina E, Tomasz A (2000) Distribution of the mosaic structured *murM* genes among natural populations of *Streptococcus pneumoniae* . J Bacteriol 182: 6798–6805. 11073926 10.1128/jb.182.23.6798-6805.2000 PMC111424

[24] Filipe SR, Severina E, Tomasz A (2002) The *murMN* operon: a functional link between antibiotic resistance and antibiotic tolerance in *Streptococcus pneumoniae* . Proc Natl Acad Sci USA 99: 1550–1555. 10.1073/pnas.032671699 11830670 PMC122228

[25] Bergmann S (2006) Versatility of pneumococcal surface proteins. Microbiology 152: 295–303. 10.1099/mic.0.28610–0 16436417

[26] Dijkstra AJ, Keck W (1996) Peptidoglycan as a barrier to transenvelope transport. J Bacteriol 178: 5555–5562. 8824596 10.1128/jb.178.19.5555-5562.1996 PMC178390

[27] Gould AR, May BK, Elliott WH (1975) Release of extracellular enzymes from *Bacillus amyloliquefaciens* . J Bacteriol 122: 34–40. 47325 10.1128/jb.122.1.34-40.1975 PMC235635

[28] Laitinen H, Tomasz A (1990) Changes in composition of peptidoglycan during maturation of the cell wall in pneumococci. J Bacteriol 172: 5961–5967. 2120197 10.1128/jb.172.10.5961-5967.1990 PMC526918

[29] Filipe SR, Severina E, Tomasz A (2001) Functional analysis of *Streptococcus pneumoniae* MurM reveals the region responsible for its specificity in the synthesis of branched cell wall peptides. J Biol Chem 276: 39618–39628. 10.1074/jbc.M106425200 11522792

[30] Hava DL, Hemsley CJ, Camilli A (2003) Transcriptional regulation in the *Streptococcus pneumoniae rlrA* pathogenicity islet by RlrA. J Bacteriol 185: 413–421. 10.1128/JB.185.2.413–421.2003 12511486 PMC145342

[31] Vollmer W, Joris B, Charlier P, Foster S (2008) Bacterial peptidoglycan (murein) hydrolases. FEMS Microbiol Rev 32: 259–286. 10.1111/j.1574-6976.2007.00099.x 18266855

[32] Briles DE, King JD, Gray MA, McDaniel LS, Swiatlo E, et al (1996) PspA, a protection-eliciting pneumococcal protein: immunogenicity of isolated native PspA in mice. Vaccine 14: 858–867. 8843627 10.1016/0264-410x(96)82948-3

[33] Guiral S, Mitchell TJ, Martin B, Claverys J-P (2005) Competence-programmed predation of noncompetent cells in the human pathogen *Streptococcus pneumoniae*: genetic requirements. Proc Natl Acad Sci USA 102: 8710–8715. 10.1073/pnas.0500879102 15928084 PMC1150823

[34] Demchick P, Koch AL (1996) The permeability of the wall fabric of *Escherichia coli* and *Bacillus subtilis* . J Bacteriol 178: 768–773. 8550511 10.1128/jb.178.3.768-773.1996 PMC177723

[35] Koch AL, Woeste S (1992) Elasticity of the sacculus of *Escherichia coli* . J Bacteriol 174: 4811–4819. 1624468 10.1128/jb.174.14.4811-4819.1992 PMC206280

[36] Merchante R, Pooley HM, Karamata D (1995) A periplasm in *Bacillus subtilis* . J Bacteriol 177: 6176–6183. 7592383 10.1128/jb.177.21.6176-6183.1995 PMC177458

[37] Matias VRF, Beveridge TJ (2005) Cryo-electron microscopy reveals native polymeric cell wall structure in *Bacillus subtilis* 168 and the existence of a periplasmic space. Mol Microbiol 56: 240–251. 15773993 10.1111/j.1365-2958.2005.04535.x

[38] Matias VRF, Beveridge TJ (2006) Native cell wall organization shown by cryo-electron microscopy confirms the existence of a periplasmic space in *Staphylococcus aureus* . J Bacteriol 188: 1011–1021. 10.1128/JB.188.3.1011–1021.2006 16428405 PMC1347357

[39] Matias VRF, Beveridge TJ (2008) Lipoteichoic acid is a major component of the *Bacillus subtilis* periplasm. J Bacteriol 190: 7414–7418. 10.1128/JB.00581-08 18790869 PMC2576656

[40] Marquis H, Hager EJ (2000) pH-regulated activation and release of a bacteria-associated phospholipase C during intracellular infection by *Listeria monocytogenes* . Mol Microbiol 35: 289–298. 10652090 10.1046/j.1365-2958.2000.01708.x PMC1763970

[41] Snyder A, Marquis H (2003) Restricted translocation across the cell wall regulates secretion of the broad-range phospholipase C of *Listeria monocytogenes* . J Bacteriol 185: 5953–5958. 14526005 10.1128/JB.185.20.5953-5958.2003 PMC225021

[42] Lemon JK, Weiser JN (2015) Degradation products of the extracellular pathogen *Streptococcus pneumoniae* access the cytosol via its pore-forming toxin. mBio 6 10.1128/mBio.02110-14 PMC4313911 25604786

[43] Atilano ML, Pereira PM, Vaz F, Catalão MJ, Reed P, et al (2014) Bacterial autolysins trim cell surface peptidoglycan to prevent detection by the Drosophila innate immune system. eLife 3 10.7554/eLife.02277.019 PMC3971415 24692449

[44] Barendt SM, Sham L-T, Winkler ME (2011) Characterization of mutants deficient in the L,D-carboxypeptidase (DacB) and WalRK (VicRK) regulon, involved in peptidoglycan maturation of *Streptococcus pneumoniae* serotype 2 strain D39. J Bacteriol 193: 2290–2300. 10.1128/JB.01555-10 21378199 PMC3133071

[45] Mellroth P, Daniels R, Eberhardt A, Ronnlund D, Blom H, et al (2012) LytA, major autolysin of *Streptococcus pneumoniae*, requires access to nascent peptidoglycan. J Biol Chem 287: 11018–11029. 10.1074/jbc.M111.318584 22334685 PMC3322828

[46] Albarracín Orio AG, Piñas GE, Cortes PR, Cian MB, Echenique J (2011) Compensatory evolution of *pbp* mutations restores the fitness cost imposed by β-lactam resistance in *Streptococcus pneumoniae* . PLoS Pathog 7: e1002000 10.1371/journal.ppat.1002000 21379570 PMC3040684

[47] Zarantonelli ML, Skoczynska A, Antignac A, Ghachi El M, Deghmane A-E, et al (2013) Penicillin resistance compromises Nod1-dependent proinflammatory activity and virulence fitness of *Neisseria meningitidis* . Cell Host Microbe 13: 735–745. 10.1016/j.chom.2013.04.016 23768497

[48] Trzciński K, Thompson CM, Gilbey AM, Dowson CG, Lipsitch M (2006) Incremental increase in fitness cost with increased β-lactam resistance in pneumococci evaluated by competition in an infant rat nasal colonization model. J Infect Dis 193: 1296–1303. 10.1086/501367 16586368

[49] Berg KH, Stamsås GA, Straume D, Håvarstein LS (2013) Effects of low PBP2b levels on cell morphology and peptidoglycan composition in *Streptococcus pneumoniae* R6. J Bacteriol 195: 4342–4354. 10.1128/JB.00184-13 23873916 PMC3807472

[50] Bui NK, Eberhardt A, Vollmer D, Kern T, Bougault C, et al (2011) Isolation and analysis of cell wall components from *Streptococcus pneumoniae* . Anal Biochem 421: 657–666. 10.1016/j.ab.2011.11.026 22192687

[51] Rijneveld AW, van den Dobbelsteen GP, Florquin S, Standiford TJ, Speelman P, et al (2002) Roles of interleukin-6 and macrophage inflammatory protein-2 in pneumolysin-induced lung inflammation in mice. J Infect Dis 185: 123–126. 10.1086/338008 11756992

[52] Orihuela CJ, Radin JN, Sublett JE, Gao G, Kaushal D, et al (2004) Microarray analysis of pneumococcal gene expression during invasive disease. Infect Immun 72: 5582–5596. 10.1128/IAI.72.10.5582–5596.2004 15385455 PMC517545

[53] Berry AM, Yother J, Briles DE, Hansman D, Paton JC (1989) Reduced virulence of a defined pneumolysin-negative mutant of *Streptococcus pneumoniae* . Infect Immun 57: 2037–2042. 2731982 10.1128/iai.57.7.2037-2042.1989 PMC313838

[54] Berry AM, Ogunniyi AD, Miller DC, Paton JC (1999) Comparative virulence of *Streptococcus pneumoniae* strains with insertion-duplication, point, and deletion mutations in the pneumolysin gene. Infect Immun 67: 981–985. 9916120 10.1128/iai.67.2.981-985.1999 PMC96416

[55] Orihuela CJ, Gao G, Francis KP, Yu J, Tuomanen EI (2004) Tissue-specific contributions of pneumococcal virulence factors to pathogenesis. J Infect Dis 190: 1661–1669. 15478073 10.1086/424596

[56] Benton KA, Everson MP, Briles DE (1995) A pneumolysin-negative mutant of *Streptococcus pneumoniae* causes chronic bacteremia rather than acute sepsis in mice. Infect Immun 63: 448–455. 7822009 10.1128/iai.63.2.448-455.1995 PMC173016

[57] Bricker AL, Camilli A (1999) Transformation of a type 4 encapsulated strain of *Streptococcus pneumoniae* . FEMS Microbiol Lett 172: 131–135. 10188240 10.1111/j.1574-6968.1999.tb13460.x

[58] Nickels BE (2009) Genetic assays to define and characterize protein-protein interactions involved in gene regulation. Methods 47: 53–62. 10.1016/j.ymeth.2008.10.011 18952173

[59] Shainheit MG, Mule M, Camilli A (2014) The core promoter of the capsule operon of *Streptococcus pneumoniae* is necessary for colonization and invasive disease. Infect Immun 82: 694–705. 10.1128/IAI.01289-13 24478084 PMC3911406

[60] Karmakar M, Katsnelson M, Malak HA, Greene NG, Howell SJ, et al (2015) Neutrophil IL-1β processing induced by pneumolysin is mediated by the NLRP3/ASC inflammasome and caspase-1 activation and is dependent on K^+^ efflux. J Immunol 194: 1763–1775. 10.4049/jimmunol.1401624 25609842 PMC4369676

[61] Avery OT, Macleod CM, McCarty M (1944) Studies on the chemical nature of the substance inducing transformation of pneumococcal types. J Exp Med 79: 137–158. 19871359 10.1084/jem.79.2.137 PMC2135445

[62] Zighelboim S, Tomasz A (1980) Penicillin-binding proteins of multiply antibiotic-resistant South African strains of *Streptococcus pneumoniae* . Antimicrob Agents Chemother 17: 434–442. 6903436 10.1128/aac.17.3.434 PMC283805

[63] Martin B, Prudhomme M, Alloing G, Granadel C, Claverys J-P (2000) Cross-regulation of competence pheromone production and export in the early control of transformation in *Streptococcus pneumoniae* . Mol Microbiol 38: 867–878. 11115120 10.1046/j.1365-2958.2000.02187.x

