# Supplementary figures and images for: Peptidoglycan Branched Stem Peptides Contribute to Streptococcus pneumoniae Virulence by Inhibiting Pneumolysin Release
Source: PLoS Pathog. 2015 Jun 26;11(6):e1004996. doi: 10.1371/journal.ppat.1004996 (PMC4483231; doi:10.1371/journal.ppat.1004996)

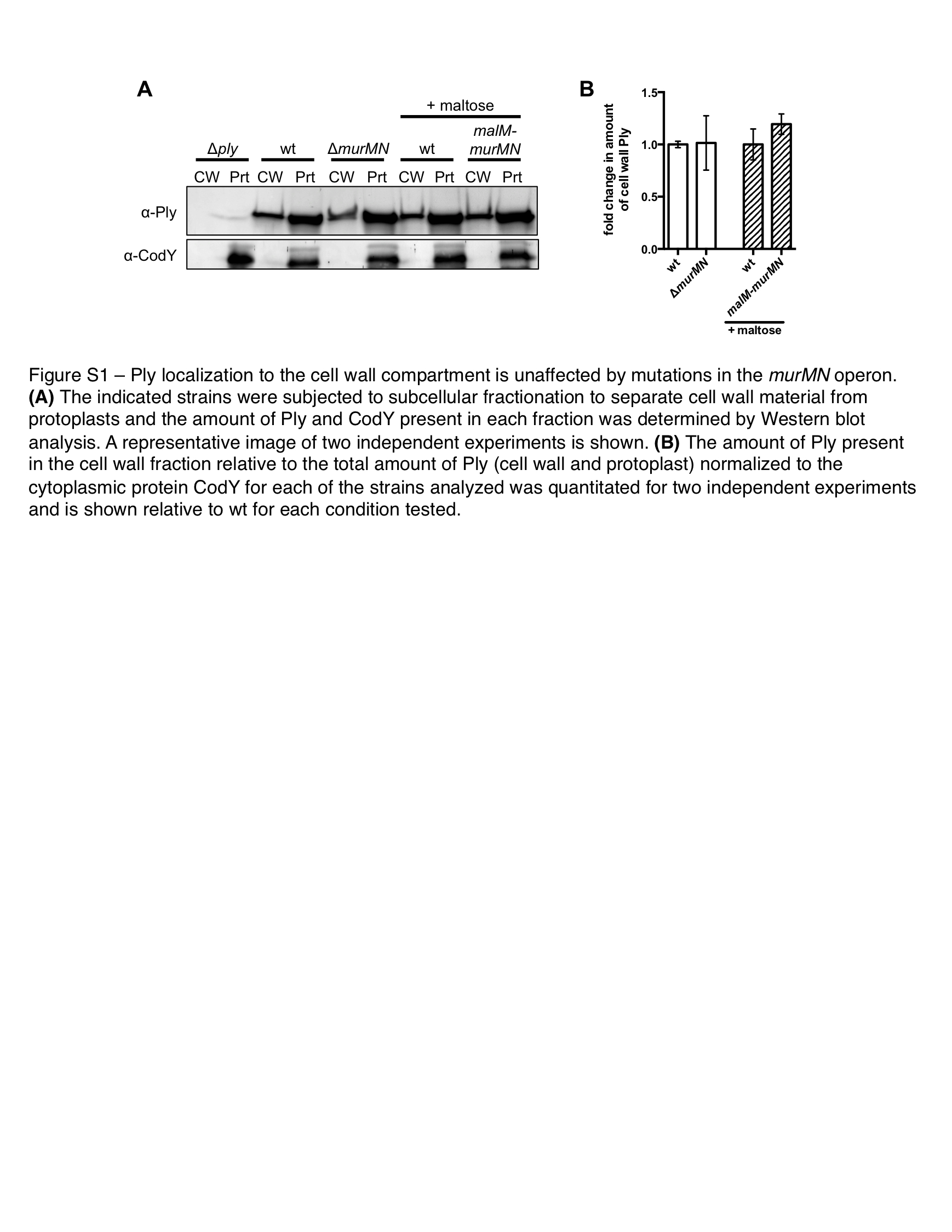

Supplement: S1 Fig — (A) The indicated strains were subjected to subcellular fractionation to separate cell wall (CW) material from protoplasts (Prt) and the amount of Ply and CodY present in each fraction was determined by Western blot analysis. A representative image of two independent experiments is shown. (B) The amount of Ply present in the cell wall fraction relative to the total amount of Ply (cell wall and protoplast) normalized to the cytoplasmic protein CodY for each of the strains analyzed was quantitated for two independent experiments and is shown relative to wt for each condition tested. (TIFF) [file ppat.1004996.s001.tiff]

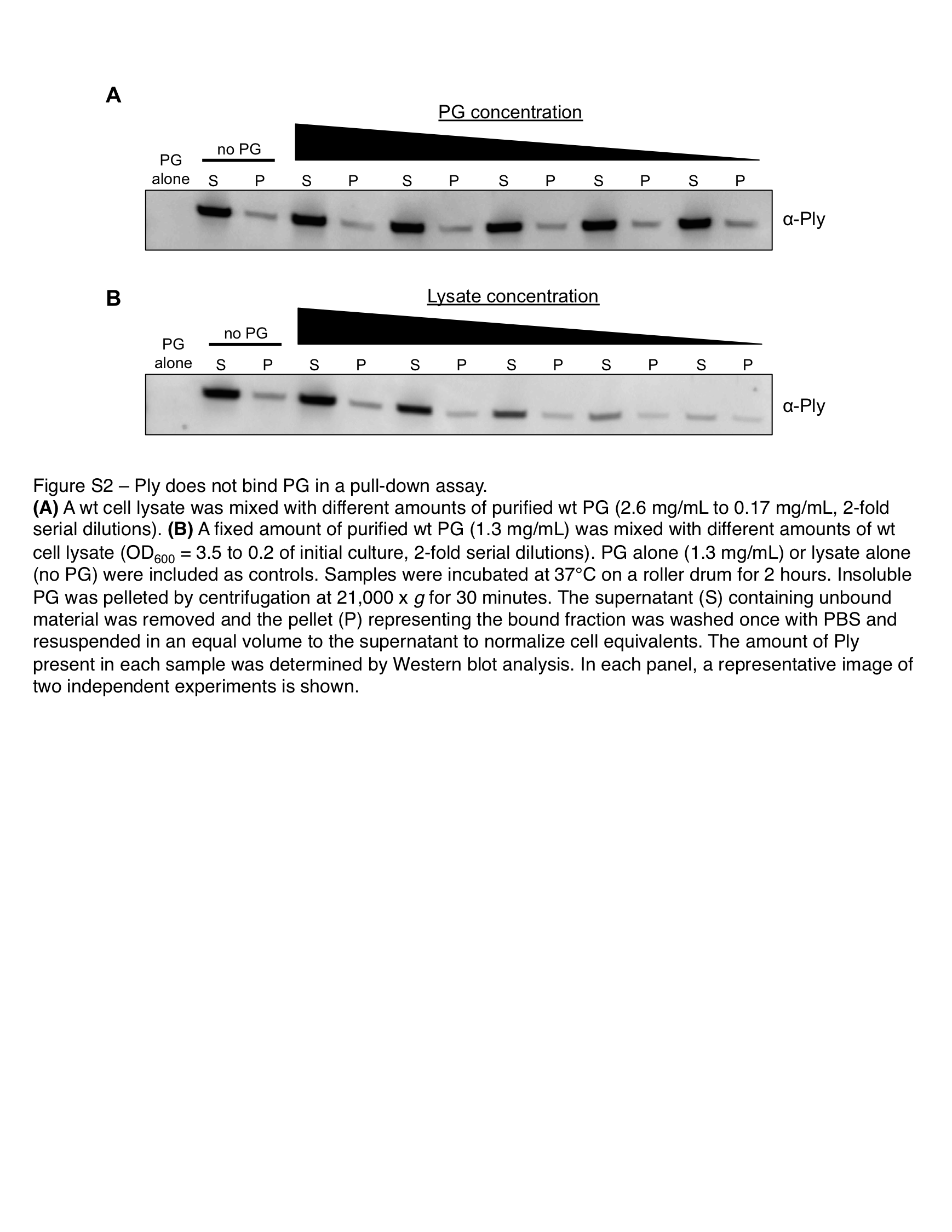

Supplement: S2 Fig — (A) A wt cell lysate was mixed with different amounts of purified wt PG (2.6 mg/mL to 0.17 mg/mL, 2-fold serial dilutions). (B) A fixed amount of purified wt PG (1.3 mg/mL) was mixed with different amounts of wt cell lysate (OD600 = 3.5 to 0.2 of initial culture, 2-fold serial dilutions). PG alone (1.3 mg/mL) or lysate alone (no PG) were included as controls. Samples were incubated at 37°C on a roller drum for 2 hours. Insoluble PG was pelleted by centrifugation at 21,000 x g for 30 minutes. The supernatant (S) containing unbound material was removed and the pellet (P) representing the bound fraction was washed once with PBS and resuspended in an equal volume to the supernatant to normalize cell equivalents. The amount of Ply present in each sample was determined by Western blot analysis. In each panel, a representative image of two independent experiments is shown. (TIFF) [file ppat.1004996.s002.tiff]

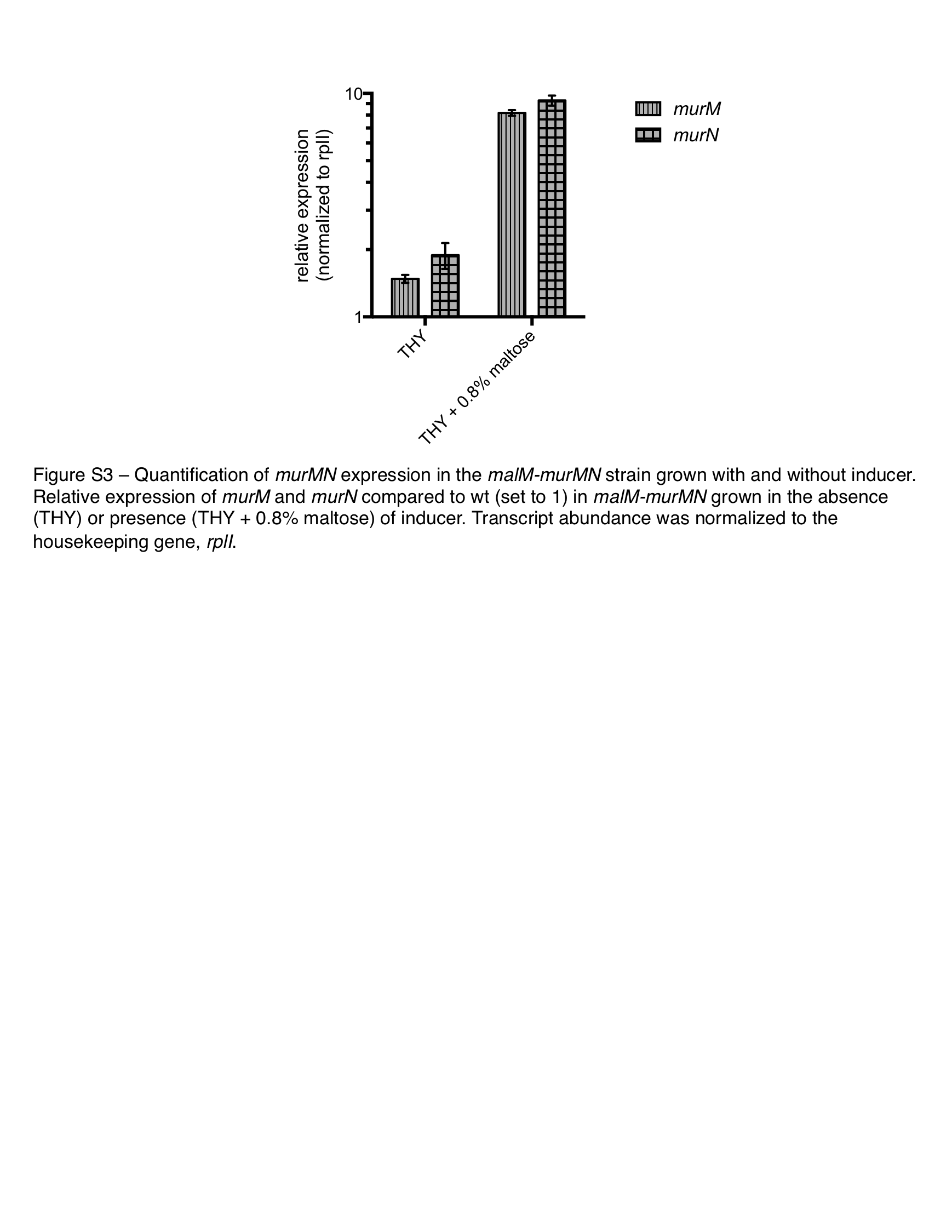

Supplement: S3 Fig — Relative expression of murM and murN compared to wt (set to 1) in malM-murMN grown in the absence (THY) or presence (THY + 0.8% maltose) of inducer. Transcript abundance was normalized to the housekeeping gene, rplI. (TIFF) [file ppat.1004996.s003.tiff]

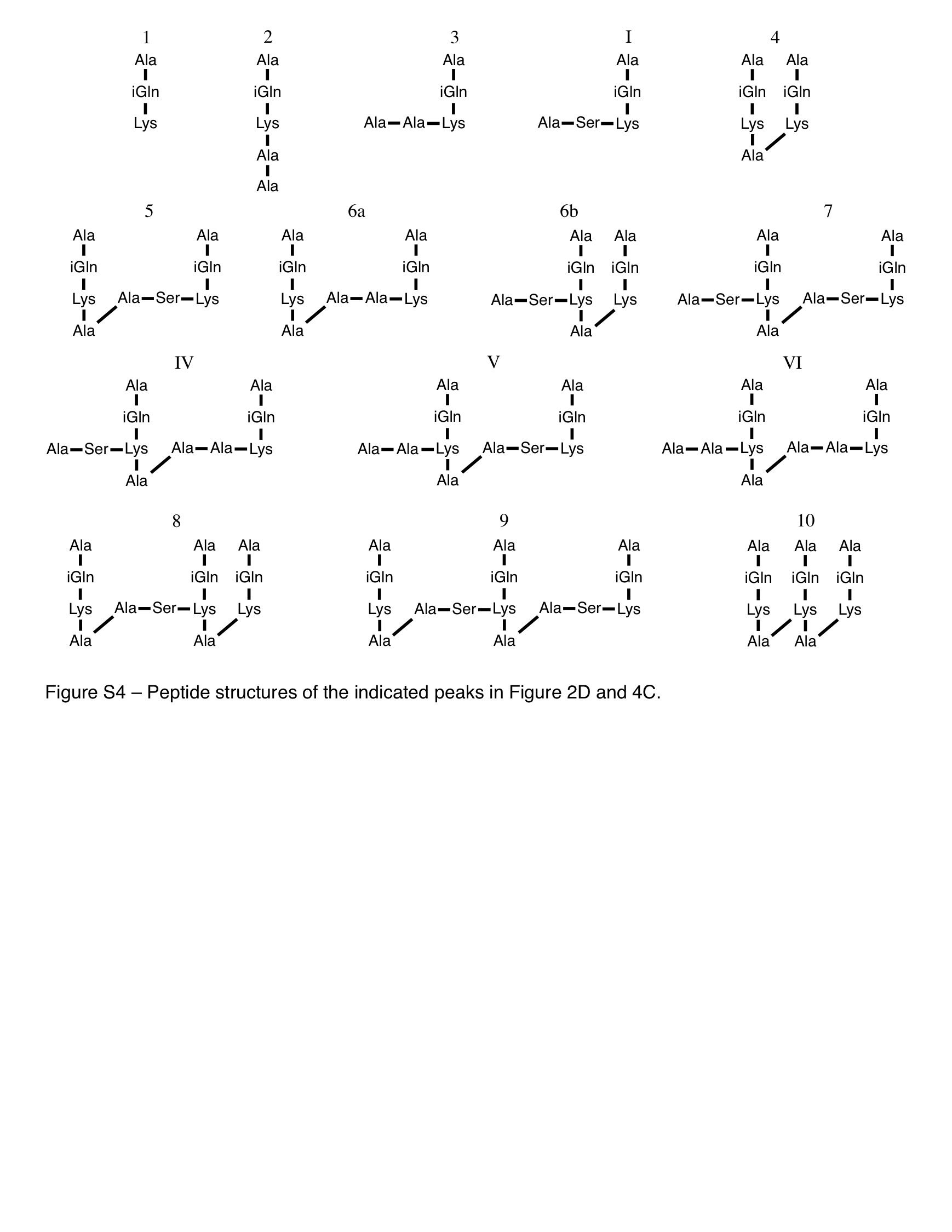

Supplement: S4 Fig — (TIFF) [file ppat.1004996.s004.tiff]

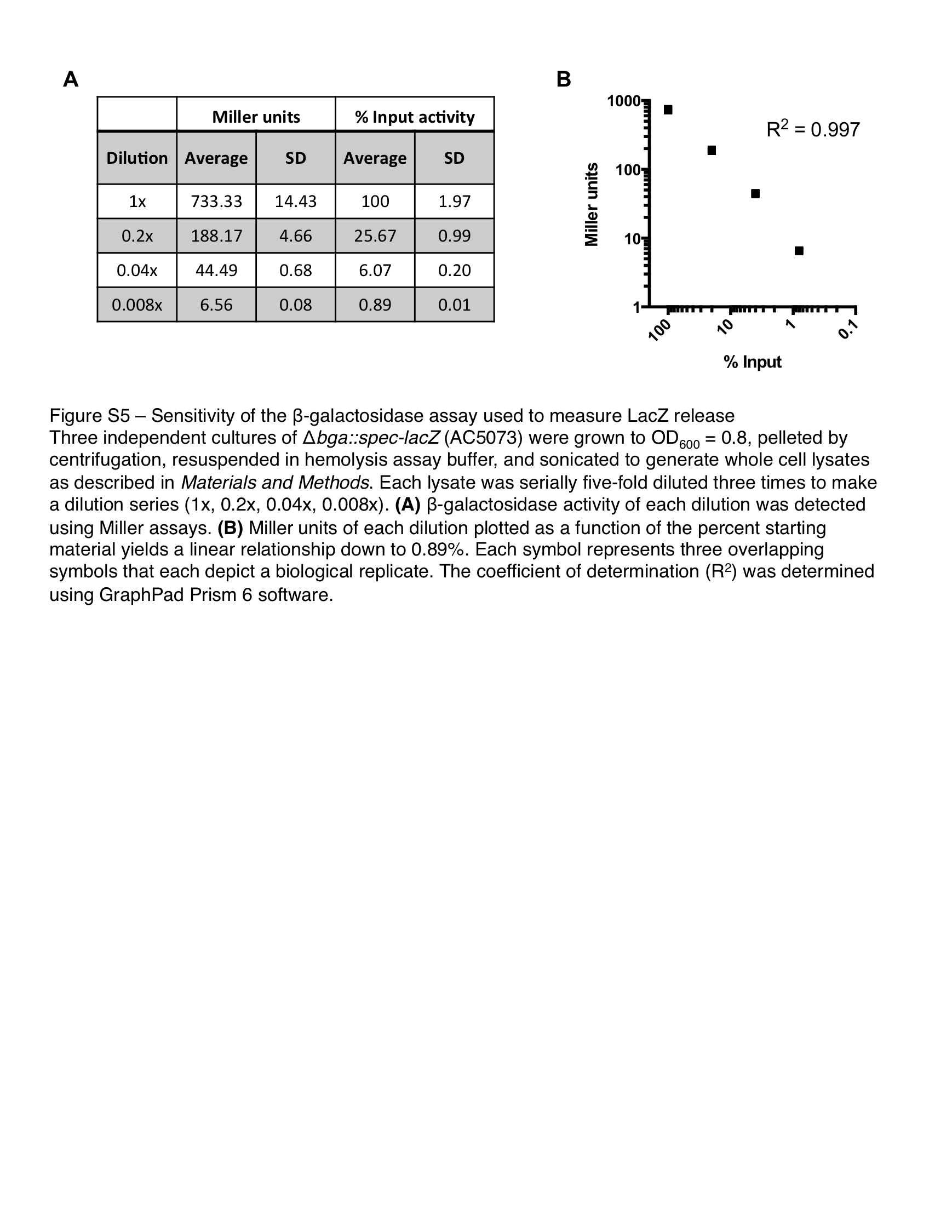

Supplement: S5 Fig — Three independent cultures of Δbga::spec-lacZ (AC5073) were grown to OD600 = 0.8, pelleted by centrifugation, resuspended in hemolysis assay buffer, and sonicated to generate whole cell lysates as described in Materials and Methods. Each lysate was serially five-fold diluted three times to make a dilution series (1x, 0.2x, 0.04x, 0.008x). (A) β-galactosidase activity of each dilution was detected using Miller assays. (B) Miller units of each dilution plotted as a function of the percent starting material yields a linear relationship down to 0.89%. Each symbol represents three overlapping symbols that each depicts a biological replicate. The coefficient of determination (R2) was determined using GraphPad Prism 6 software. (TIFF) [file ppat.1004996.s005.tiff]
